# Machine learning from quantum chemistry to predict experimental solvent effects on reaction rates†

**DOI:** 10.1039/d3sc05353a

**Published:** 2024-01-10

**Authors:** Yunsie Chung, William H. Green

**Affiliations:** a Department of Chemical Engineering, Massachusetts Institute of Technology Cambridge MA 02139 USA whgreen@mit.edu

## Abstract

Fast and accurate prediction of solvent effects on reaction rates are crucial for kinetic modeling, chemical process design, and high-throughput solvent screening. Despite the recent advance in machine learning, a scarcity of reliable data has hindered the development of predictive models that are generalizable for diverse reactions and solvents. In this work, we generate a large set of data with the COSMO-RS method for over 28 000 neutral reactions and 295 solvents and train a machine learning model to predict the solvation free energy and solvation enthalpy of activation (ΔΔ*G*^‡^_solv_, ΔΔ*H*^‡^_solv_) for a solution phase reaction. On unseen reactions, the model achieves mean absolute errors of 0.71 and 1.03 kcal mol^−1^ for ΔΔ*G*^‡^_solv_ and ΔΔ*H*^‡^_solv_, respectively, relative to the COSMO-RS calculations. The model also provides reliable predictions of relative rate constants within a factor of 4 when tested on experimental data. The presented model can provide nearly instantaneous predictions of kinetic solvent effects or relative rate constants for a broad range of neutral closed-shell or free radical reactions and solvents only based on atom-mapped reaction SMILES and solvent SMILES strings.

## Abstract 2

A machine learning model, trained on a large COSMO-RS dataset, enables accurate and rapid predictions of solvation effects on reaction rates for diverse reactions and solvents only based on atom-mapped reaction SMILES and solvent SMILES.

## Graphical abstract

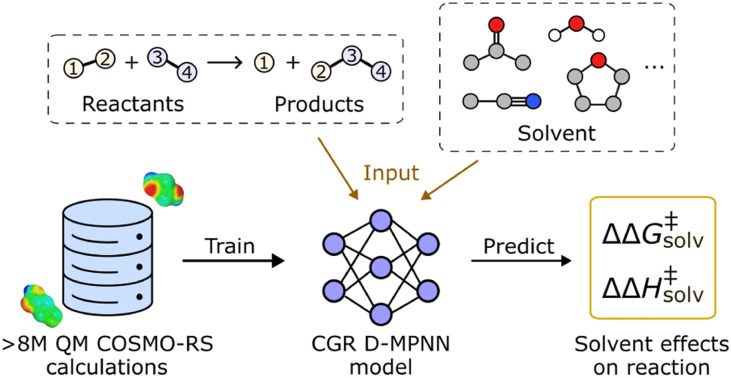

## 1 Introduction

Accurate prediction of reaction rates is essential for modeling a variety of chemical kinetic systems such as pyrolysis,^1,2^ polymerization,^3^ oxidative degradation,^4,5^ and atmospheric chemistry.^6^ Detailed kinetic models enable one to predict key products, identify major kinetic pathways, and optimize reaction conditions for complex chemical systems. Kinetic mechanisms often involve hundreds to tens of thousands of elementary reactions,^7^ and a fast, high-throughput method to estimate reaction rates is thus needed. *Ab initio* methods like quantum mechanics/molecular mechanics (QM/MM) can provide accurate predictions of rate constants, but their high computational cost has been a major limiting factor for large-scale, automated predictions. As more kinetic data become available, data-driven approaches such as linear group contribution,^8–10^ decision tree based rate rules,^11,12^ and machine learning (ML) models^13–19^ have emerged as more popular choices for estimating kinetic parameters. Several ML models^15–17^ have successfully predicted barrier heights and rate constants of diverse gas phase reactions only based on readily available 2D information (*e.g.* SMILES strings) of reactants and products. However, such data-driven models for liquid/solution phase reactions have been lightly investigated with limited applicability,^20^ and most approaches rely on the *ab initio* methods with either implicit or explicit solvation models.^21,22^

Solvents can have significant impacts on reaction rates and outcomes, and it is crucial to accurately predict these kinetic solvent effects. Recent research efforts have been devoted to employing ML (*e.g.* deep neural network) for free energy predictions of condensed phase reactions.^15,18,19,23–28^ Many of these studies^18,19,23–25,28^ combine the ML models with semi-empirical or lower-level QM/MM methods to obtain the energy predictions that match the accuracy of higher-level QM/MM methods. For example, Gómez-Flores *et al.*^19^ used a ML approach to predict the energy difference between the density functional tight-binding model and other higher level QM methods for a thiol-disulfide exchange reaction in water. In a study by Pan *et al.*,^18^ a ML model was trained to reproduce *ab initio* QM/MM potentials in free energy simulations for the aqueous Menshutkin reaction between ammonia and chloromethane. Farrar and Grayson^28^ employed ML models to predict DFT-quality activation barriers for various nitro-Michael addition reactions in toluene based on the features generated from semi-empirical methods. These approaches, however, require semi-empirical QM/MM steps that are less suitable for instantaneous, automatic rate predictions. Furthermore, their models are limited to a single solvent and need the 3D coordinates or QM features of reactants and transition states as inputs, which are not readily available.

The ML models by Jorner *et al.*^26^ and by Heid and Green^15^ are the few cases that can predict reaction properties in multiple solvents only based on the 2D structural information of molecules. Jorner *et al.*^26^ employed a Gaussian process regression model and compared several 2D structural features to predict the barrier height of 443 S_N_Ar reactions in different solvents. In their work, the best accuracy was reached by adopting the BERT^29^ reaction fingerprint. Heid and Green,^15^ on the other hand, used the condensed graph of reaction (CGR) as an input reaction representation for a graph convolutional neural network (GCNN). They applied the CGR GCNN model to the same S_N_Ar data set and were able to achieve better barrier height predictions compared to the other models that used the BERT fingerprint or different reaction representations. While these models can provide fast kinetic estimations for solution-phase reactions at a low computational cost, only one reaction family was considered with a relatively small training set. A larger data set that contains more diverse types of reactions and solvents is needed in order to train a more generalized model for kinetic solvent effect predictions. Moreover, both models used fixed descriptors to represent solvents, but prior studies^15,30,31^ revealed that the learned molecular representations based on a graph convolutional approach outperform fixed molecular descriptors in many property prediction tasks.

In this study, we present a ML model that can predict kinetic solvent effects for a wide range of neutral reactions and solvents only based on atom-mapped reaction SMILES and solvent SMILES strings. More precisely, the model predicts the solvation free energy and solvation enthalpy of activation (ΔΔ*G*^‡^_solv_, ΔΔ*H*^‡^_solv_) for a reaction–solvent pair, which can be used to estimate a relative rate constant between a solution phase and a gas phase reaction or between the reaction in different solvents. Our model adopts a CGR GCNN architecture with separate GCNN layers for solvent molecular encoding. A large, diverse set of training data containing over 28 000 reactions and 295 solvents is generated in this work by performing *ab initio* COSMO-RS^32^ calculations. The performance of the model on unseen reactions is rigorously assessed by comparing the ML predictions with both COSMO-RS calculations and experimental data. A transfer learning approach and various additional features are explored to further improve the model. Our ML model can provide accurate predictions of relative rate constants, and together with the existing predictive models or databases for gas phase rate constants (*e.g.* RMG database^12^), it can provide the estimates of absolute rate constants for many different liquid phase reactions.

## 2 Background on the prediction targets

Our ML model aims to predict the solvation free energy and solvation enthalpy of activation (ΔΔ*G*^‡^_solv_, ΔΔ*H*^‡^_solv_) at 298 K for a reaction in a solvent. Solvation free energy (Δ*G*_solv_) and solvation enthalpy (Δ*H*_solv_) are the changes in Gibbs free energy and enthalpy when a molecule is transferred from an ideal gas to a solvent at a fixed condition. The ΔΔ*G*^‡^_solv_ and ΔΔ*H*^‡^_solv_ of a reaction–solvent pair are defined as the solvation free energy and solvation enthalpy differences between a transition state (TS) and reactant(s):

1 ΔΔ*G*^‡^_solv_ = Δ*G*^TS^_solv_ − Δ*G*^R^_solv_

2 ΔΔ*H*^‡^_solv_ = Δ*H*^TS^_solv_ − Δ*H*^R^_solv_

where Δ*G*^TS^_solv_ and Δ*G*^R^_solv_ represent the solvation free energies of a TS and a reactant, and Δ*H*^TS^_solv_ and Δ*H*^R^_solv_ represent the solvation enthalpies of a TS and a reactant, respectively. For a bimolecular reaction, Δ*G*^R^_solv_ and Δ*H*^R^_solv_ each correspond to the sum of the solvation free energies and solvation enthalpies of all reactants. The standard state of 1 M ideal gas and 1 M solution is used for solvation free energy and enthalpy in this work.

**Fig. 1:**
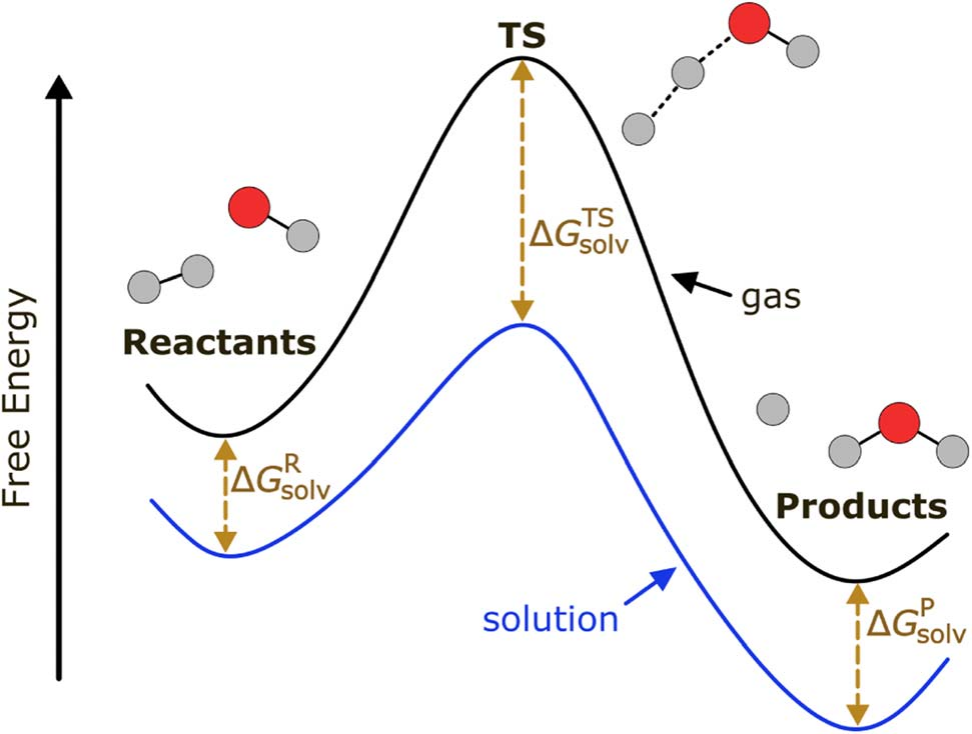
Potential energy diagram of a reaction in a gas phase and a solution phase.

As depicted in Fig. 1, a solvent medium can affect the energies of reactants and a TS by different degrees, causing the activation free energy to shift when a reaction occurs in a solution (liquid) phase. The ΔΔ*G*^‡^_solv_ of a reaction corresponds to the difference in the free energy of activation between a gas phase and a solution phase and is an important kinetic parameter for solution phase reactions. For example, ΔΔ*G*^‡^_solv_ can be directly used to estimate the ratio of a gas phase rate constant (*k*_gas_) to a liquid phase rate constant (*k*_liq_) as follows:^33^3
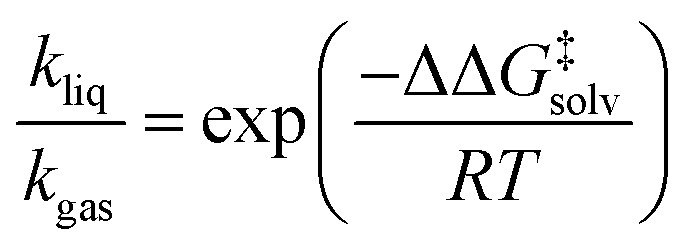
where *R* is the universal gas constant and *T* is a temperature. It can be also used to calculate the relative rate constant between two solvents:4
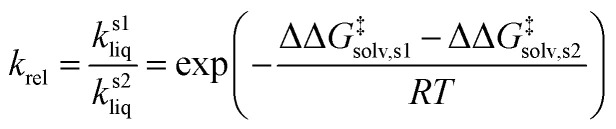
where *k*^s1^_liq_ and *k*^s2^_liq_ are the rate constants of a reaction in a solvent 1 and in a solvent 2, respectively, and ΔΔ*G*^‡^_solv,s1_ and ΔΔ*G*^‡^_solv,s2_ are the corresponding solvation energies of activation for the reaction in each solvent.

Our model predicts ΔΔ*H*^‡^_solv_ in addition to ΔΔ*G*^‡^_solv_ at 298 K to account for the temperature dependence of ΔΔ*G*^‡^_solv_. The ΔΔ*G*^‡^_solv_ at a different temperature can be linearly extrapolated using the two model outputs at 298 K as follows:5
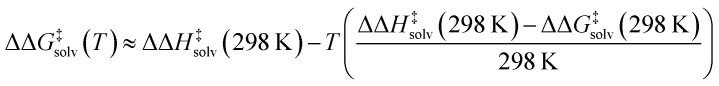


The linear approximation is found to be generally valid for a moderate temperature range (250–400 K),^34^ but the error is expected to increase as the temperature significantly deviates from 298 K.

## 3 Methods

### 3.1 Data generation

Table 1 shows the summary of the data sets used in this work. A total of three data sets are prepared: (1) a pre-training set containing the reactions from Grambow *et al.*,^35–37^ (2) a fine-tuning set containing the reactions from Harms *et al.*,^38^ and (3) an experimental test set from our prior study.^33^ The data sets include diverse range of neutral closed-shell and free radical reactions and nonionic solvents. For both pre-training and fine-tuning sets, ΔΔ*G*^‡^_solv_ and ΔΔ*H*^‡^_solv_ are computed for each reaction–solvent pair with the COSMO-RS calculations based on the geometries obtained from Grambow *et al.* and Harms *et al.* The ML model is trained, validated, and tested on the computed data, and the experimental set is used as an additional test set for the final error assessment. The reaction and solvent information is stored as atom-mapped reaction SMILES and solvent SMILES in all data sets.

**Table 1:** Summary of the data sets used in this study. The number of reactions in the pre-training and fine-tuning sets include both forward and reverse directions. “*N* data chosen” represents the number of data sampled from the total data to construct the training, validation, and test sets

Data set	*N* data total	*N* data chosen	*N* reactions	*N* solvents	Data type & reference
Pre-training set	7 796 583	500 000 (6.4%)	26 448	295	In-house COSMO-RS calculations based on the optimized geometries from Grambow *et al.*^35–37^
Fine-tuning set	542 833	46 122 (8.5%)	1870	295	In-house COSMO-RS calculations based on the optimized geometries from Harms *et al.*^38^
Experimental test set	165	165	15	49	Experimental relative rate constants from Chung and Green^33^

We separated the computed data into the pre-training and fine-tuning sets because the two data sets significantly differ in the types of reactions included and the level of theory used for geometry optimizations. The pre-training set is the largest, but the majority of its reactions are uncommon reactions with high gas phase barrier heights (*e.g. E*_a_ > 50 kcal mol^−1^), and it does not contain any reactions that are bimolecular in both forward and reverse directions (*e.g.* only A → B, A + B → AB, and AB → A + B reactions appear). In contrast, the fine-tuning set is smaller but contains more common reactions. To leverage the different types of data, we employ a transfer learning approach in which the model is first pre-trained on the reactions from Grambow *et al.* and subsequently fine-tuned on the reactions from Harms *et al.* Details on each data set and the computational method are described below, and all data sets are provided through Zenodo (https://zenodo.org/record/8423911).

#### 3.1.1 Computational method

The pre-training and fine-tuning data sets are generated by performing COSMO-RS calculations at the BP86/def2-TZVPD^39–41^ level of theory with fine grid cavity,^42^ which is commonly known as a BP-TZVPD-FINE level. The COSMO-RS is a hybrid solvation model that uses quantum chemistry and statistical thermodynamics to compute the chemical potential of a compound in a solvent.^32,43,44^ We have previously demonstrated that the COSMO-RS method can provide accurate predictions of ΔΔ*G*^‡^_solv_ for various neutral closed-shell and free radical reactions in different solvents with a mean absolute error of around 0.45 kcal mol^−1^.^33^

The computational workflow used in this work follows that employed in our earlier study.^33^ Single-point energy calculations are performed at the BP-TZVPD-FINE level of theory in a COSMO phase and in a gas phase with TURBOMOLE 7.5 (ref. 45 and 46) for reactants, products, and TSs based on the optimized gas phase geometries obtained from Grambow *et al.* and Harms *et al.*; this step generates screening charge densities and energies that are needed for the COSMO-RS calculations. Then, the Δ*G*_solv_ and Δ*H*_solv_ of reactants, products, and TSs are computed in 295 common solvents at 298 K with COSMOtherm (release 2021)^47^ using the BP_TZVPD_FINE_21 parametrization based on the COSMO-RS theory, and the ΔΔ*G*^‡^_solv_ and ΔΔ*H*^‡^_solv_ of each reaction are subsequently calculated in 295 solvents at 298 K using eqn (1) and (2). Because COSMOtherm does not directly output solvation enthalpy, ΔΔ*H*^‡^_solv_ is obtained by first computing ΔΔ*G*^‡^_solv_ at 297, 298, and 299 K, estimating the temperature gradient at 298 K, and then using the definition 
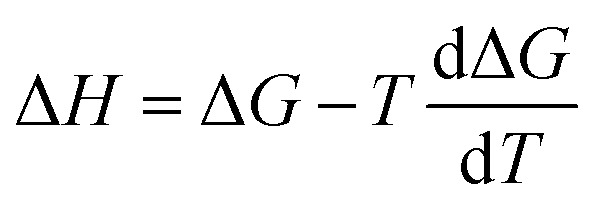
. Our prior studies showed that the proposed way can yield accurate approximations of solvation enthalpy and these approximated values together with eqn (5) can give accurate estimates of solvation free energy at a temperature range of 250–400 K.^34,48^ The screening charge densities and energies of the 295 solvents are acquired from the COSMObase database.^49^ The reactions from Grambow *et al.* and Harms *et al.* were each optimized in gas phase at the ωB97XD3/def2-TZVP^50,51^ and M06-2X/cc-pVTZ^52,53^ levels of theory in their original work. Although these levels of theory are different from the level used for the COSMO-RS calculations, our prior work^33^ demonstrated that the accurate COSMO-RS calculations can be made with the gas phase geometries that are optimized at different levels of theory, which justifies the current computational workflow.

A total of 7 814 610 and 614 780 COSMO-RS calculations were completed successfully for the pre-training and fine-tuning sets, respectively. The results were then further cleaned by only including the reaction–solvent pairs that successfully ran for both forward and reverse directions of the reaction. While most of the computed ΔΔ*G*^‡^_solv_ and ΔΔ*H*^‡^_solv_ values were within ±10 kcal mol^−1^, a small fraction of the data had unreasonably large values for neutral reactions. For instance, a ΔΔ*G*^‡^_solv_ of ±40 kcal mol^−1^ corresponds to around 29 orders of magnitude increase/decrease in a liquid phase rate constant compared to a gas phase rate constant (see eqn (3)). We suspected that these are likely due to the COSMO-RS calculation errors as the COSMO-RS method may not have been parameterized well for certain reactions and geometries. Therefore, we filtered out 241 reaction–solvent pairs from the pre-training set that had |ΔΔ*G*^‡^_solv_| > 40 kcal mol^−1^ or |ΔΔ*H*^‡^_solv_| > 56 kcal mol^−1^ (14 standard deviations away from means). Higher quality data are usually expected for the fine-tuning set. Thus, more strict cutoff values of |ΔΔ*G*^‡^_solv_| > 10 kcal mol^−1^ and |ΔΔ*H*^‡^_solv_| > 18 kcal mol^−1^ are applied to the fine-tuning set to remove potentially erroneous data.

#### 3.1.2 Pre-training set

The final pre-training set contains a total of 7 796 583 reaction–solvent pairs with 26 448 unique reactions and 295 solvents. Both forward and reverse reactions are included in the data set to augment the data. As mentioned earlier, the geometry optimizations were done at the ωB97XD3/def2-TZVP level of theory for these reactions in the original work by Grambow *et al.*^35–37^ The histograms and statistics of the data set are provided in ESI Fig. S1.† The computed ΔΔ*G*^‡^_solv_ and ΔΔ*H*^‡^_solv_ have nearly normal distributions with high peaks at zero. The ΔΔ*G*^‡^_solv_ and ΔΔ*H*^‡^_solv_ have absolute mean values of 1.81 and 2.58 kcal mol^−1^, respectively, with standard deviations of 2.76 and 3.92 kcal mol^−1^. The reactions contain diverse types of neutral closed-shell and free radical reactions that involve H, C, N, and O atoms and have at most 7 heavy atoms. Due to errors from Open Babel^54^ when perceiving connectivity, a small set of the original reaction SMILES from the Grambow *et al.* had incorrect bond orders and formal charges, and therefore, the corrected atom-mapped SMILES from Spiekermann *et al.*^55^ are used for our pre-training set.

The entire data set has nearly 7.8 million data points. However, it is unlikely that every reaction–solvent pair is needed since the total number of unique reactions and solvents would remain fixed even if the number of reaction–solvent pairs increases. To investigate the effect of the data size on the model performance, we prepared 8 different data sets containing 10k, 50k, 75k, 100k, 250k, 500k, 750k, and 1m data points. These data are sampled in a semi-random manner such that all reactions and solvents appear in the data sets at least once, except the 10k set which has fewer data than the total number of reactions. From the results, we determined 500k to be the optimal data set size for the model as explained further in the Results section.

#### 3.1.3 Fine-tuning set

The fine-tuning data set has 542 833 reaction–solvent pairs with 1870 unique reactions including both forward and reverse directions and 295 solvents. The geometry optimizations were performed at the M06-2X/cc-pVTZ level of theory in the original work by Harms *et al.*^38^ for these reactions. The data set contains three specific reaction types: bimolecular hydrogen abstraction (H-abstraction), unimolecular hydrogen migration (H-migration), and radical addition to a multiple bond (R-addition). These are neutral, free radical reactions that are ubiquitous in both gas and liquid phase systems. The reaction templates are illustrated in the ESI Fig. S2.† In total, there are 1402 H-abstraction, 146 H-migration, and 322 R-addition reactions. The reactions involve maximum 10 heavy atoms and include H, C, and O atoms. The histograms and statistics of the data are presented in ESI Fig. S3.† The ΔΔ*G*^‡^_solv_ and ΔΔ*H*^‡^_solv_ have absolute average values of 1.40 and 2.32 kcal mol^−1^, respectively, with standard deviations of 1.91 and 3.17 kcal mol^−1^.

Similar to the pre-training set, only a subset of the fine-tuning data was chosen for the model. We sampled around 25 solvents per each reaction semi-randomly with more weights on polar solvents in order to include more data with stronger solvent effects. A total of 46 122 data points were selected, and all reactions and solvents appear in the chosen set at least once. The detailed data sampling method is explained in ESI Section S2.†

#### 3.1.4 Experimental test set

The experimental data set^33^ consists of 165 relative rate constants (*k*_rel_) for 15 neutral reactions and 49 solvents from 273 K to 392 K. None of the experimental reactions appear in the pre-training and fine-tuning sets, and therefore these data serve as a reaction split test set for the final model evaluation. The reactions are depicted in ESI Table S1,† and they include 2 β-scission, 5 H-abstraction, 3 Diels–Alder, and 5 other types of reactions. The reactions involve H, C, N, O, and S atoms and have up to 22 heavy atoms, which are much larger than the reactions found in the pre-training and fine-tuning sets. The atom mappings of these reactions are obtained using the tool from ref. 56, and incorrect atom mappings are then manually fixed. The errors on the experimental test set are computed in both log_10_(*k*_rel_) and Δ*G*^‡^_rel_ units, where the Δ*G*^‡^_rel_ error is calculated as follows:

6 Δ*G*^‡^_rel_ error = −*RT*(ln(*k*_rel,expt_) − ln(*k*_rel,calc_))

### 3.2 Data splits

The pre-training set is split into a 90% training/validation and a 10% test set using reaction and solvent splits to evaluate the model's predictive performance on unseen reactions and unseen solvents. For our test splits, 5% reactions and 5% solvents are randomly selected, and all reaction–solvent pairs that include the chosen reactions or chosen solvents are added to the test set and excluded from the training/validation set. Both forward and reverse directions of the selected reactions are included in the test set to prevent data leakage; this procedure is very crucial for evaluating the true performance of a model on unseen reactions since the model can gain the information on the reaction and TS from its own reverse reaction. The importance of the proper reaction split is addressed in the recent studies by Heid and Green^15^ and Spiekermann *et al.*^16^ The remaining 90% data are randomly split into a 80% training and 20% validation set. The validation set is used for early stopping to determine the epoch that gives the lowest validation error and prevents over-fitting. Five folds are used to prepare five different training, validation, and test sets from the pre-training set.

The fine-tuning set is randomly split into a 80% training and a 20% validation set using one fold. We did not construct a separate test set from the fine-tuning set. Instead, the experimental set is used as a final reaction-split test set for the fine-tuned model. We ensured that none of the experimental reactions (both forward and reverse) appear in the pre-training and fine-tuning sets.

### 3.3 Machine learning model architecture

**Fig. 2:**
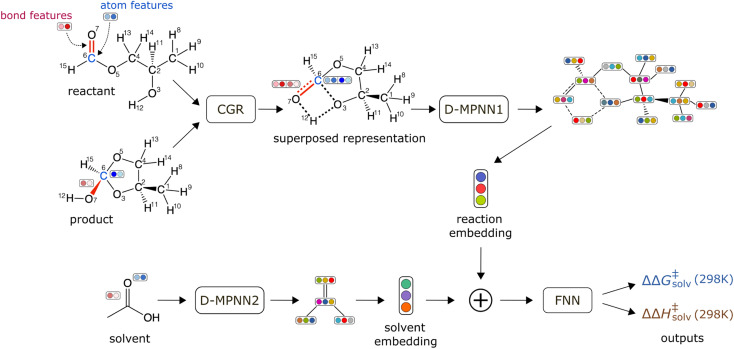
Schematic of a machine learning model architecture. The model takes an atom-mapped reaction SMILES and a solvent SMILES as inputs.

The schematic of the ML architecture is depicted in Fig. 2. Our model takes the atom-mapped reaction SMILES and solvent SMILES as inputs and predicts ΔΔ*G*^‡^_solv_ and ΔΔ*H*^‡^_solv_ at 298 K. The model is constructed using Chemprop,^30,57^ an open-source software that uses a directed message passing neural network (D-MPNN) for chemical and reaction property predictions. A D-MPNN is a type of GCNN that converts atom and bond features into a latent representation of a molecule through bond-level message passing. To encode a reaction, we adopt the established CGR representation^15,58,59^ as it has shown to outperform other representations for various reaction property predictions. The CGR is a superposition of the reactant and product graphs, which mimics the 2D-structure of the TS. In our model, the CGR representation is constructed from the atom-mapped reactants and products and passed into a D-MPNN to give a reaction embedding. A separate D-MPNN is employed to convert a solvent graph into a solvent molecular embedding. The learned reaction and solvent representations are then concatenated together and passed into a feed forward neural network (FNN) to predict the regression targets. The initial atom and bond features are generated using RDKit^60^ within Chemprop and include several features such as atom type, bond type, formal charge, chirality, and *etc.* The hyperparameters are optimized *via* 47 iterations of Bayesian optimization with the Hyperopt package.^61^ Only the training/validation set of the pre-training data is used for the hyperparameter optimization to prevent data leakage. The full list of atom and bond features and the optimized hyperparameters can be found in ESI Tables S2 and S3.†

As mentioned in the earlier section, a transfer learning approach is used to first train the model on the pre-training set and subsequently fine-tune the model on the fine-tuning set with fewer epochs. Within each of the 5 folds of the pre-training set, an ensemble of 5 different models are generated by using different random initialization of model parameters. This results in a total of 25 individual models from the 5-fold pre-training set. The optimized parameters from the 25 pre-trained models are then used to initialize the 25 fine-tuned models that are trained on the fine-tuning set. When evaluating the reaction- and solvent-split errors on the pre-training test set, average predictions from the ensemble of 5 models are employed to compute the error in each fold. When evaluating the final error on the experimental set, an ensemble of all 25 fine-tuned models are used to make the average predictions. We ensured that no models are trained or validated on the tested reactions or solvents.

### 3.4 Additional features

Five additional sets of features that are listed in Table 2 are explored to improve the model performance. The RP-solv features represent the solvation free energies and solvation enthalpies of reactants and products that are calculated in this work. We also tested the Abraham parameters of solvents (S-abr) that are obtained from the ML model by Chung *et al.*^48^ The Abraham parameters consist of five descriptors that can describe various solute/solvent interactions.^64^ The RDKit-mol represents the 2D molecular features generated from RDKit. There are 200 2D molecular features available within RDKit, and 20 and 15 features were selected for reactants/products and solvents, respectively, based on the variance threshold and random forest methods imported from scikit-learn.^65^ Six additional RDKit atomic features (Addit-atom) that are adopted from Vermeire and Green^62^ are also tested as they have shown to improve solvation free energy predictions. These include the number of radical electrons, ring size, number of lone electron pairs, H-bond donating and accepting characters, and electronegativity. Lastly, the QM atomic and bond descriptors (QM-desc) obtained from the ML model by Guan et el.^63^ are explored. The QM-desc contains 4 atomic descriptors (Hirshfeld partial charge, two Fukui indices, NMR shielding constants) and 2 bond descriptors (bond lengths, bond orders) that were shown to improve reaction property predictions.^63,66^

**Table 2:** List of additional features investigated

Name	Type	Description	Ref.
RP-solv	Molecular	Solvation energy and enthalpy of reactants and products at 298 K (Δ*G*^R^_solv_, Δ*G*^P^_solv_, Δ*H*^R^_solv_, Δ*H*^P^_solv_)	This work
S-abr	Molecular	Abraham parameters of solvents	48
RDKit-mol	Molecular	2D molecular features generated from RDKit	60
Addit-atom	Atomic	Additional atom features generated from RDKit	60 and 62
QM-desc	Atomic, bond	QM atom and bond descriptors	63

The performances of the additional features are compared using the pre-training test set. Within the ML model, additional molecular features are concatenated with the reaction and solvent embeddings and fed into the FFN layer to make the predictions. Additional atom and bond features are concatenated with the initial atom and bond features prior to the CGR/D-MPNN layers. Note that nearly all features can be calculated instantly or predicted by existing ML models. The only exception is the RP-solv features which are computed with the COSMO-RS method. Yet, several ML models are available for predicting solvation energy and enthalpy of closed-shell compounds,^48,62,67–70^ and the RP-solv features can be therefore estimated with the ML models if fast approximations are needed. We did not consider the 3D structures of the reactants and products as additional inputs in our study as they are usually not readily available and prone to calculation noise and error. Furthermore, Spiekermann *et al.*^16^ showed that the 2D D-MPNN model outperformed the 3D ML model for gas phase barrier height predictions on the Grambow *et al.*'s reactions. Since the same data set and similar model architecture are used in our study, we expect the result to be similar and hence do not consider the 3D ML model in this work.

## 4 Results and discussion

### 4.1 Data set size and additional features

**Fig. 3:**
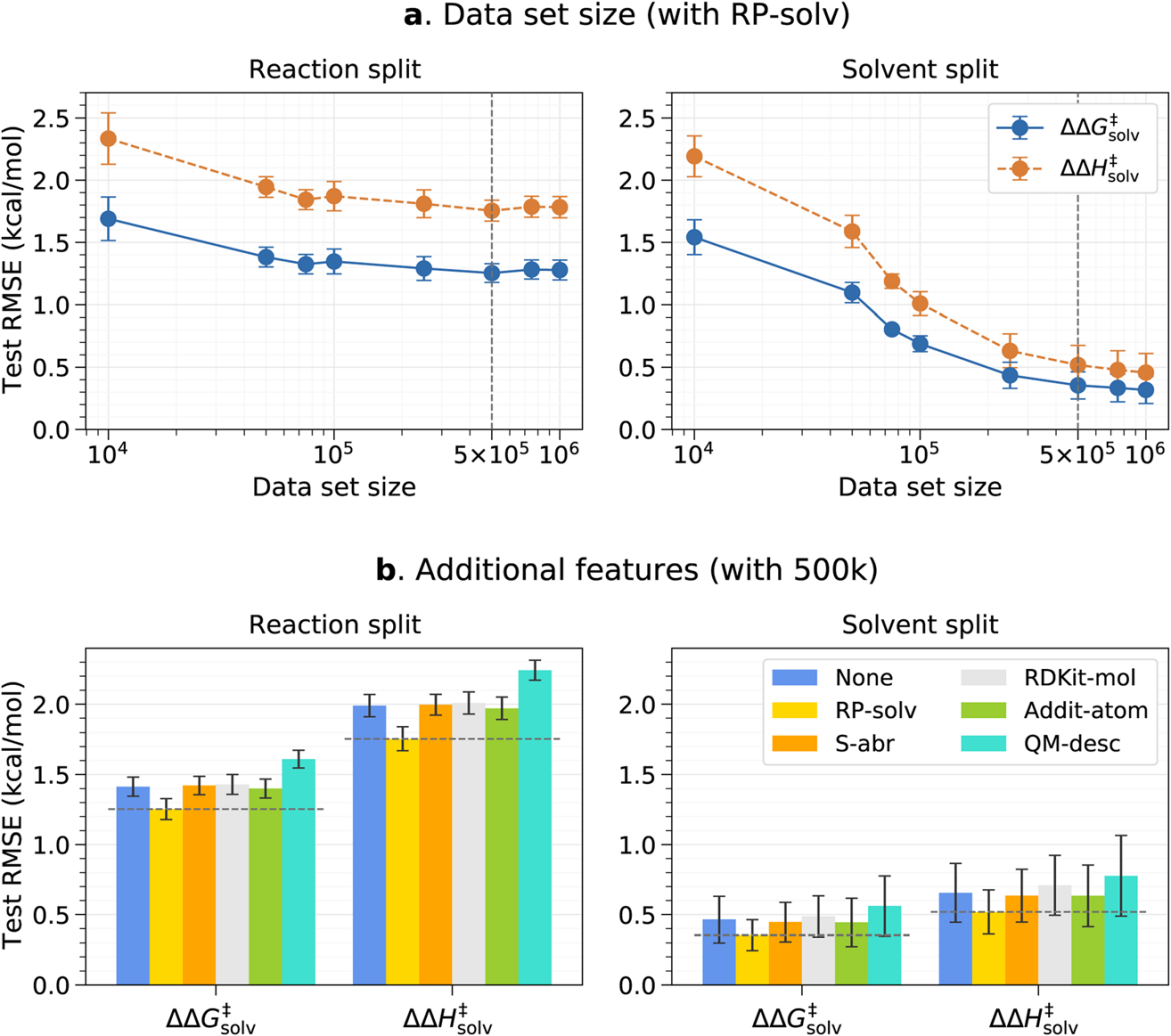
Pre-trained model results on the reaction split and solvent split test sets. (a) RMSE *vs.* the data set size for the model trained with the RP-solv feature. (b) RMSE error of different additional features for the model trained with the 500k data set. The chosen data set size and feature are marked with dashed vertical and horizontal lines, respectively. The error bars indicate the standard deviation between five folds.

The effects of the data set size and additional features are investigated using the pre-training set prior to fine-tuning any models. The resulting test root-mean-square errors (RMSE) on the reaction and solvent splits are presented in Fig. 3. The reaction and solvent splits each test the model's performance on unseen reactions in seen solvents and on seen reactions in unseen solvents. As previously explained, the pre-training set of around 7.8m data is divided into smaller subsets to identify the optimal data set size that can balance accuracy and training time. From Fig. 3a, it can be seen that the test error initially decreases with an increasing data set size and plateaus out from 500k for the reaction split. For the solvent split, the error continues to decrease at a higher data set size, but the change in the error is very small beyond 500k. Therefore, 500k is chosen as a final data set size for the pre-training set. This result was expected since only the number of reaction–solvent pairs increases with the increasing data set size whereas the number of unique reactions and solvents remains constant. The information gain from more reaction–solvent pairs is likely to saturate after the model sees enough data on each reaction and solvent, causing the errors to level out.

Fig. 3b shows the results of the additional features tested with the 500k data set. In all cases, only the RP-solv feature improves the errors and other features do not have noticeable impacts on the model performance. The reactions tend to accelerate/decelerate in a polar solvent when the products are more/less strongly solvated than the reactants.^71,72^ The RP-solv feature, which consists of solvation energy and solvation enthalpy of reactants and products, describe how strongly the reactants and products are solvated in a solvent and therefore helps the ΔΔ*G*^‡^_solv_ and ΔΔ*H*^‡^_solv_ predictions for our model. The results also show that the QM-desc feature worsens the model performance, which is consistent with results from Spiekermann *et al.*^16^ that show the descriptors offer no improvement. The QM-desc feature was predicted by the ML model from Guan *et al.*^63^ which was trained on three classes of substitution reactions. Our data set includes more diverse types of reactions, and therefore, it is likely that their model is not suitable for our reaction data. Moreover, it is possible that the chosen QM descriptors are not related to our targets and act as noise to the model.

### 4.2 Comparison of the pre-trained and fine-tuned models

Table 3 summarizes the performances of the pre-trained and fine-tuned models with and without the RP-solv feature. The MAE (mean absolute error) and RMSE are reported in kcal mol^−1^, and the standard deviations are calculated between the 5 folds for the pre-training set. For the experimental test set, the *k*_rel_ error is converted to a Gibbs free energy unit (Δ*G*^‡^_rel_ or *RT*(ln *k*_rel_)) using eqn (6) to allow easy comparison.

**Table 3:** Test errors of different models and additional (addit.) features. The best combination of the model and additional feature is marked in bold. For the pre-training set, the errors on the reaction split are available for all models while the errors on the solvent split are only available for the pre-trained model and shown in parentheses. The standard deviations are calculated between the 5 folds for the pre-trained set. All test errors are reported in kcal mol^−1^

Model	Addit. feature	Pre-training set: reaction split (solvent split)				Experimental set	
		ΔΔ*G*^‡^_solv_		ΔΔ*H*^‡^_solv_		Δ*G*^‡^_rel_	
		MAE	RMSE	MAE	RMSE	MAE	RMSE
Pre-trained	None	0.79 ± 0.03 (0.20 ± 0.05)	1.41 ± 0.07 (0.46 ± 0.17)	1.13 ± 0.04 (0.30 ± 0.07)	1.99 ± 0.08 (0.66 ± 0.21)	0.89	1.14
Pre-trained	RP-solv	0.68 ± 0.02 (0.16 ± 0.04)	1.25 ± 0.07 (0.35 ± 0.11)	0.97 ± 0.03 (0.24 ± 0.06)	1.75 ± 0.08 (0.52 ± 0.16)	0.73	0.95
**Fine-tuned**	**None**	**0.71 ± 0.14** (—)	**1.16 ± 0.24** (—)	**1.03 ± 0.18** (—)	**1.63 ± 0.33** (—)	**0.68**	**0.88**
Fine-tuned	RP-solv	0.82 ± 0.08 (—)	1.29 ± 0.18 (—)	1.40 ± 0.09 (—)	2.06 ± 0.21 (—)	0.70	0.90

The fine-tuned model with no additional feature achieves overall the best performance on unseen reactions for both pre-training and experimental test sets. The model has the *RT*(ln *k*_rel_) MAE/RMSE of 0.68/0.88 kcal mol^−1^ on the experimental set and has the ΔΔ*G*^‡^_solv_ and ΔΔ*H*^‡^_solv_ MAE/RMSE of 0.71/1.16 and 1.03/1.63 kcal mol^−1^, respectively, on the pre-training set reaction split. The model has higher ΔΔ*H*^‡^_solv_ errors than ΔΔ*G*^‡^_solv_ in all cases as the COSMO-RS method, which was used to generate the training data, has higher calculation errors for ΔΔ*H*^‡^_solv_.^48^ Furthermore, ΔΔ*H*^‡^_solv_ generally has a larger magnitude than ΔΔ*G*^‡^_solv_, which leads to larger absolute errors. The results also show that the pre-trained model has much lower errors on the solvent split than the reaction split. The model is able to provide very accurate predictions on unseen solvents with 295 training solvents whereas it has much higher errors on unseen reactions even with 26 448 training reactions. We believe this is because the chemical space of viable solvents is not as nearly big as that of reactions. Furthermore, the reaction split is a more challenging task since the model has to infer the TS information from the reactants and products. The fine-tuned model was not separately tested on the solvent split as it was trained on all solvents that are found in the fine-tuning set. Since the major limitation is on the reaction split, we expect the fine-tuned model to have a similarly low error on unseen solvents.

Contrary to the earlier results on the pre-trained model, it is found that the RP-solv feature does not improve the fine-tuned model. Upon closer examination, we observed that the fine-tuned model with the RP-solv feature has lower training and validation loss than the model without the feature, but has higher error on both pre-training and experimental test sets. The discrepancy in performance suggests that the model overfits to the RP-solv feature during fine-tuning. The fine-tuning set contains only three classes of reactions, which are more common reactions but are less diverse than the pre-training set. It appears that the information learned about the RP-solv feature during fine-tuning does not generalize well to other reaction classes. In contrast, the fine-tuned model without the feature performs better by avoiding overfitting and also benefits from a reduced computational cost as it no longer requires the RP-solv features that need to be calculated for each reaction–solvent pair.

Table 3 shows that the best fine-tuned model achieves around 0.1–0.2 kcal mol^−1^ lower error than the pre-trained model on the unseen experimental reactions. The performance gain is relatively big considering the small size of the fine-tuning set compared to the size of the pre-training set. The fine-tuning set contains bimolecular reactions that the pre-training set lacks and includes more common classes of reactions while the pre-training set largely contains uncommon reactions with high gas phase barrier heights (*E*_a_ > 50 kcal mol^−1^).^35^ Hence, even a relatively small number of fine-tuning data greatly enhances the model's performance on the experimental set, which mostly contains low-barrier reactions and several bimolecular reactions. A similar result was observed in the work by Spiekermann *et al.*^16^ where a model that was initially pre-trained with lower accuracy DFT data showed substantial improvement on barrier height predictions after fine-tuning with a small number of higher accuracy CCSD(T)-F12 data. Both their and our studies demonstrate that different types of data sets can be best leveraged *via* transfer learning when only a limited amount of higher quality or more relevant data is available. Transfer learning is particularly beneficial for our study since we could avoid mixing the two data sets that differ in the level of theory used for geometry optimizations and also put more emphasis on the data set that is considered to be more relevant to real liquid phase systems.

It is also worthwhile to note that the model has similar or slightly lower errors on the pre-training set reaction split after fine-tuning. Even though the pre-training and fine-tuning sets differ in the level of theory used for geometry optimizations and the types of reactions included, fine-tuning improves the model's performance on the pre-training test set as well. Our prior study^33^ demonstrated that the ΔΔ*G*^‡^_solv_ calculations using the COSMO-RS method are not too sensitive to the level of theory used for geometry optimizations for the 15 experimental reactions tested. Similar conclusion can be deduced from the current result as the fine-tuning set, which is based on the M06-2X/cc-pVTZ geometries, still helps or does not exacerbate the model's predictions on the pre-training set, which is based on the ωB97XD3/def2-TZVP geometries.

**Fig. 4:**
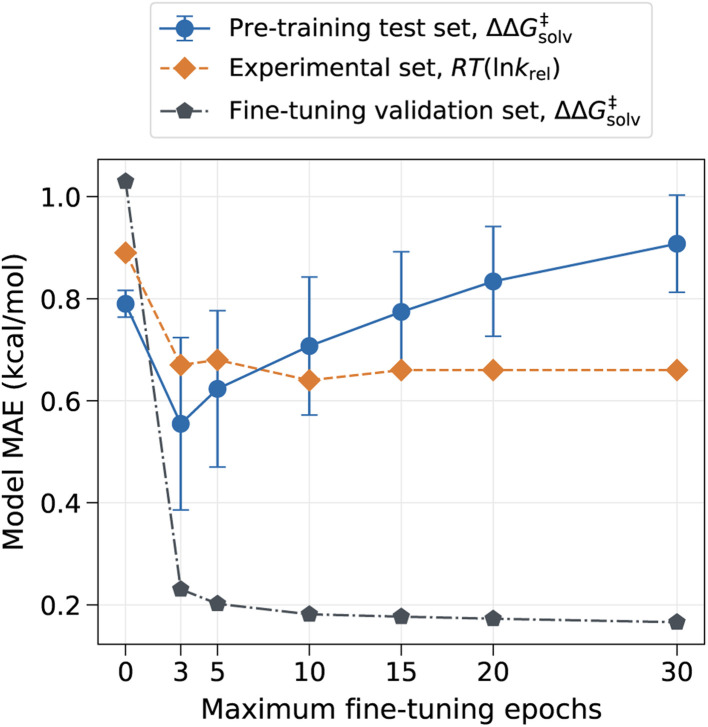
Model MAE *vs.* the number of fine-tuning epochs. The model is trained with no additional feature. The error on the pre-training set is evaluated on the reaction split test set, and the error on the fine-tuning set is evaluated on the random split validation set.

However, the model can have drastically different outcomes depending on the number of fine-tuning epochs used. In this work, the ML model was trained up to the chosen number of maximum epochs, and the final model was selected based on the best validation loss. We used the maximum epoch of 80 for pre-training and used the smaller maximum epoch of 10 for fine-tuning to prevent the pre-trained information from being completely overwritten by the three reaction families used in the fine-tuning set. The fine-tuning epoch was set to 10 because the validation error plateaued out after 10 epochs, as depicted in Fig. 4. Fig. 4 shows that the error on the pre-training set initially has a sharp drop as the model learns new reactions but gradually increases as the number of maximum fine-tuning epochs increases. The error on the experimental set also decreases at first but soon levels off at around 10 epochs. The result indicates that the chosen epoch maintains a good balance between retaining previously learnt knowledge and learning new data. At higher epochs, however, the model starts to lose prior knowledge without much added benefits as it becomes biased toward the three reaction families found in the fine-tuning set. It is thus important to identify optimal epochs and hyperparameters for the fine-tuned model if one seeks to preserve the pre-trained information.

**Fig. 5:**
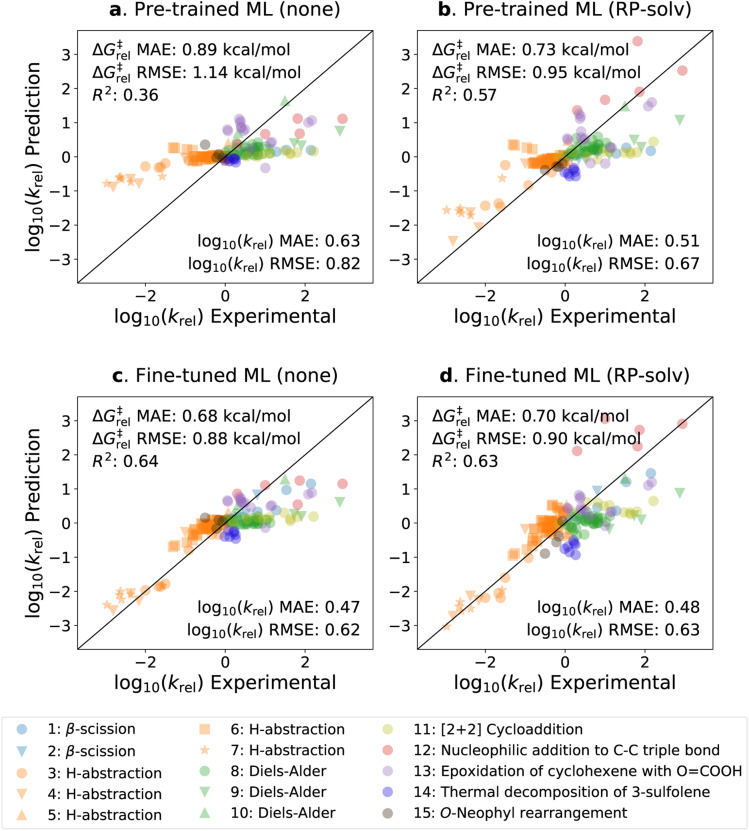
Parity plots of the predicted *vs.* experimental *k*_rel_ values. (a) The pre-trained model with no additional feature. (b) The pre-trained model with the RP-solv feature. (c) The fine-tuned model with no additional feature. (d) The fine-tuned model with the RP-solv feature. The errors are reported in both log_10_(*k*_rel_) and Δ*G*^‡^_rel_ units, and *R*^2^ represents the coefficient of determination.

The parity plots of the pre-trained and fine-tuned models are presented in Fig. 5 for the experimental test set. It can be seen that the predictions on the H-abstraction reactions are substantially improved after fine-tuning the model. This was expected as the fine-tuning set primarily comprises H-abstraction reactions. The fine-tuned models also have slightly improved predictions on β-scission reactions. The models, on the contrary, have relatively poor performance on Reactions 9, 11, 12, and 13 (see ESI Table S1† for details on the reactions). Notably, the fine-tuned model with the RP-solv feature exhibits high deviations for Reaction 12,† which is a nucleophilic addition reaction. Our former study^33^ revealed that while varying the level of theory had little impact on log_10_(*k*_rel_) calculations for most reactions, Reaction 12† displayed particularly high sensitivity to the levels of theory used in calculations. Therefore, it is possible that the RP-solv features (Δ*G*_solv_ and Δ*H*_solv_ of reactants and products) calculated for Reaction 12† were not accurate enough and led to higher errors.

Considering that the sizes of reactants and TSs in the experimental set are approximately twice as large as those in the training sets, the model demonstrates satisfactory extrapolation capability after fine-tuning. Specifically, when no additional features are used, the pre-trained model predicts the log_10_(*k*_rel_) values to be close to zero for nearly all reactions, whereas the fine-tuned model shows noticeable enhancement in capturing the trend. We expect the model to improve further as more diverse and larger reactions become available for training. It is also worth highlighting that the model was trained with the calculated data, which inherently carry some degrees of error. We previously found that the COSMO-RS method has a Δ*G*^‡^_rel_ MAE/RMSE of around 0.4 kcal mol^−1^ (0.28 in log_10_*k*_rel_ units) on these reactions.^33^ This implies that only a part of the prediction errors are attributed to the model's performance, and the rest stems from the intrinsic calculation errors within the training data.

**Fig. 6:**
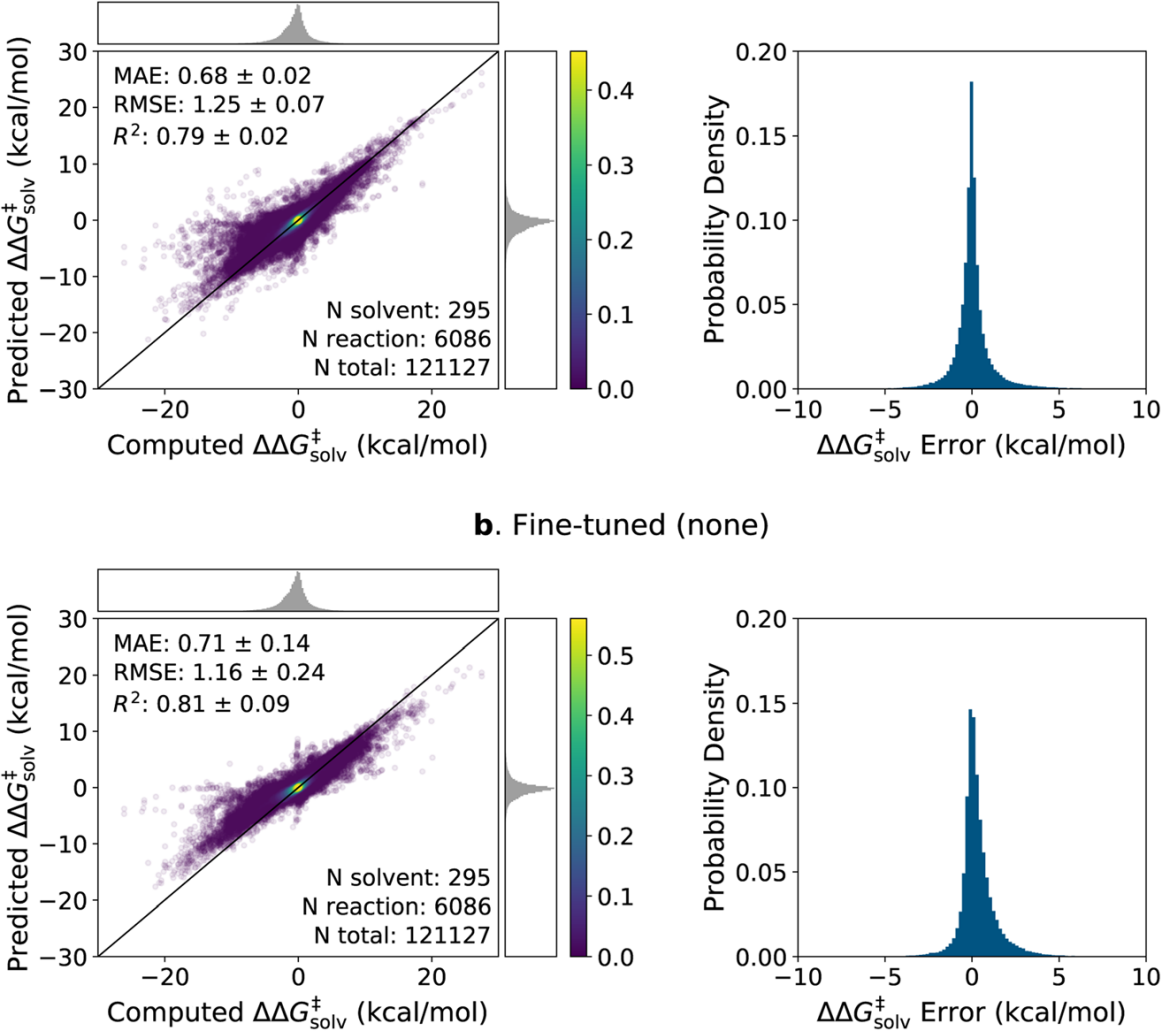
Parity plots and histograms of the ΔΔ*G*^‡^_solv_ errors on the 5-fold pre-training set reaction split. (a) The pre-trained model with the RP-solv feature. (b) The fine-tuned model with no additional feature. The MAE and RMSE are in kcal mol^−1^. The numbers of reactions, solvents, total data points found in the test set are provided. The top and right subfigures on the parity plots show the distribution of computed and predicted values, and the colorbars display the scale of the 2D kernel density estimate plots.

The parity plots and error histograms of the best pre-trained and fine-tuned models are provided in Fig. 6 for the ΔΔ*G*^‡^_solv_ predictions on the pre-training set reaction split. The corresponding plots for all models on the ΔΔ*H*^‡^_solv_ predictions and for the solvent split are presented in ESI Fig. S4–S6.† The test errors are centered around zero for both models, and the majority of the errors fall within ±2 kcal mol^−1^. Yet, higher errors are observed in regions where the data are sparse. The pre-trained model predicts nearly zero ΔΔ*G*^‡^_solv_ values for many reaction–solvent pairs whose computed ΔΔ*G*^‡^_solv_ values are highly negative. Such trend is less pronounced in the fine-tuned model, but the model still tends to underpredict the magnitude of the ΔΔ*G*^‡^_solv_ values when the computed ΔΔ*G*^‡^_solv_ have large positive or negative values. It is important to clarify, though, that the model is compared with the computed values and not with the true values. The ΔΔ*G*^‡^_solv_ of ±10 kcal mol^−1^ corresponds to around 7 orders of magnitude difference between the liquid phase and gas phase rate constants at room temperature. Such large solvent effects are very rare for neutral reactions, and thus, it is possible that the COSMO-RS method overpredicted the magnitude of the target values on some of these extreme data points.

**Fig. 7:**
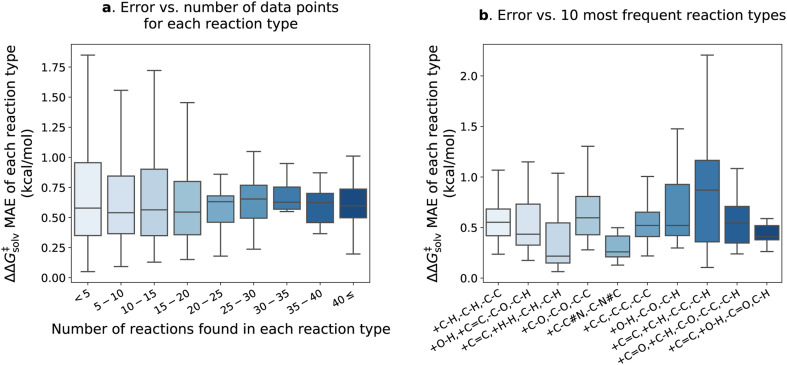
The results of the fine-tuned model with no additional feature on the pre-training test set reaction split. (a) Distribution of the ΔΔ*G*^‡^_solv_ MAE categorized by the number of training reaction data found in each reaction type. (b) Distribution of the ΔΔ*G*^‡^_solv_ MAE for the 10 most frequent reaction types. The reaction type is specified by the bond changes. For example, +C–H, –C–H, –C–C indicates that one carbon–hydrogen bond is formed, one carbon–hydrogen bond is broken, and one carbon–carbon bond is broken. Outliers are not shown in the plots.

Fig. 7 delves into the results of the fine-tuned model. The reactions within the pre-training set are categorized into different types based on bond changes to investigate potential variations in test error across reaction types. The pre-training set comprises 4476 reaction types identified for 13 224 reactions (considering only forward directions), with 1571 of these reaction types found in the reaction split test set. Approximately 90% of the reaction types have fewer than 5 reactions matching each type and are mostly unique. In Fig. 7a, the ΔΔ*G*^‡^_solv_ errors of reaction types are plotted against the number of the corresponding reactions in each type. The error distribution appears larger for the reaction types with fewer than 20 reactions, but the mean error values remain similar across all ranges. The test set error distributions of the 10 most frequent reaction types are also examined in Fig. 7b. The examples of the reactions corresponding to the 10 types are provided in the ESI Table S5.† The results show that the +C=C, +C–H, –C–C, –C–H type exhibits the highest mean error of 0.88 kcal mol^−1^. However, the error is still close to the overall ΔΔ*G*^‡^_solv_ MAE of the fine-tuned model. It appears that a few outliers contribute to the higher errors of the +C=C, +C–H, –C–C, –C–H type (see Table S5†), but it is not obvious which chemical functionalities are associated with higher errors in these reactions. Table S5† reveals that the reactions within the same type are also diverse and unique, making it challenge to establish a clear correlation between reaction type and prediction error. This observation aligns with the findings of Grambow *et al.*,^13^ who also did not see a clear correlation between reaction type and test set error in their study on predicting activation barriers.

We further examined the outliers of the fine-tuned model on the pre-training test set reaction split. The top 20 reactions with the highest test errors are given in ESI Table S4† along with their gas phase barrier heights obtained from Grambow *et al.*^35–37^ It is found that the majority of the outliers are unusual reactions such as those forming biradical products, involving TSs with high ring strain, and with high barrier heights (*E*_a_ > 85 kcal mol^−1^). These reactions are unlikely to occur in real condensed phase systems, and therefore we anticipate the model to have lower errors on more feasible reactions.

**Fig. 8:**
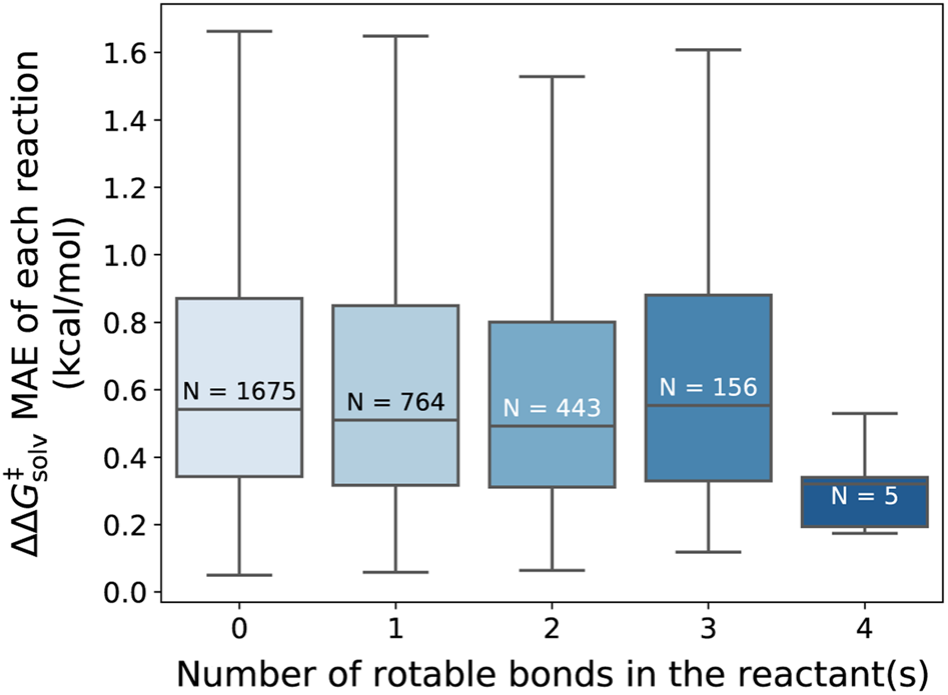
Distribution of the ΔΔ*G*^‡^_solv_ MAE categorized by the number of rotatable bonds found in the reactant(s) of each reaction (outliers not shown). The *N* indicates the number of reactions in each distribution. The fine-tuned model without additional feature tested on the pre-training set reaction split.

Overall, our model gives reliable predictions of solvent effects on numerous neutral reactions. The model is easy to use as only reaction and solvent SMILES are needed without requiring any additional computational steps. However, it should be highlighted that the proposed method is constructed based on some assumptions. We assume that the solvation effect does not change the 3D geometries of the reactant and TS. The training set is generated by performing single-point energy calculations in a solvent with the geometry of the lowest-energy conformer optimized in the gas phase, as provided by the original work of Grambow *et al.* and Harms *et al.*, and potential conformational changes upon solvation were not considered. While prior studies^33,73^ indicate that the assumption generally gives acceptable predictions, conformer effects can be very crucial for the reactions involving compounds with many rotatable bonds, or zwitterions/tautomers and can lead to higher prediction errors. Fig. 8 illustrates the distribution of the ΔΔ*G*^‡^_solv_ errors per the number of rotatable bonds found in the reactant(s). While there is no clear correlation between the test error and the number of rotatable bonds, it can be seen that the majority of the reactants are rigid, with no or less than 4 rotatable bonds. Thus, future studies should focus on generating more thorough training set that contains more flexible and larger compounds and should consider various conformers of reactants, products, and TSs.

It is also essential to note that the effect of solvent friction has been neglected. Our ML model predicts the solvent effects on the activation barriers, but diffusion control should be taken into account if the predicted reaction rate is above the diffusion limit for a bimolecular reaction. Moreover, the majority of the reactions in the training set are unimolecular in a either forward or reverse direction (*e.g.* A → B, A + B → AB, and AB → A + B), and the bimolecular H-abstraction in the fine-tuning set is the only reaction family that are bimolecular in both forward and reverse directions. We assume the learning of unimolecular systems can also provide good approximations for bimolecular systems. However, the model could have larger predictions errors for a new class of bimolecular reactions, and caution should be made for such reactions.

## 5 Conclusions

We developed a machine learning model that can provide fast and accurate predictions of kinetic solvent effects for a wide range of neutral reactions and solvents. A large set of training data were generated using the COSMO-RS method for over 28 000 reactions and 295 solvents. The performance of the model was evaluated with both calculated and experimental data using rigorous data splits. The model achieves the MAEs of 0.71 and 1.03 kcal mol^−1^ on unseen reactions for the prediction of solvation free energy and solvation enthalpy of activation (ΔΔ*G*^‡^_solv_, ΔΔ*H*^‡^_solv_), respectively, relative to the COSMO-RS calculations. The model is shown to provide reliable predictions of relative rate constants when tested on the experimental set that contains unseen reactions with much bigger molecules than those found in the training set. We also demonstrate that different types of data sets can be effectively used *via* a transfer learning approach to refine the predictions.

The presented model can be used to estimate the relative rate constants between a gas phase and a liquid phase or between two solvents for a temperature range of around 250 K to 400 K. If a rate constant in one solvent or in a gas phase is known for a reaction, our model outputs can be used to estimate absolute rate constants in many different solvents for a given reaction. One of the advantages of the model is that it only needs the atom-mapped reaction SMILES and solvent SMILES as inputs, which are more amenable for automatic high-throughput predictions in comparison to requiring optimized 3D geometries as input. We anticipate the model to be particularly useful for the design of chemical processes and automatic construction of reaction mechanisms where fast estimations of kinetic parameters and solvent screenings are needed for a large number of reaction–solvent pairs.

## Data availability

All data sets and the fine-tuned ML model can be found through Zenodo: https://zenodo.org/record/8423911. A sample script for making ΔΔ*G*^‡^_solv_ and ΔΔ*H*^‡^_solv_ predictions with the fine-tuned ML model can be found at https://github.com/yunsiechung/chemprop/tree/RxnSolvKSE_ML. The data sets and model are open access and distributed under the terms and conditions of the Creative Commons Attribution (CC BY 4.0) license (https://creativecommons.org/licenses/by/4.0/). The details on the data sets, model hyperparameters, parity plots of all model predictions, prediction outliers, and examples of reaction types are provided in the ESI.†

## Author contributions

Y. C. conceived the project, performed the simulations, prepared the data sets, wrote the computer code, and wrote the manuscript. W. H. G. provided project administration and funding acquisition, supervised the research, and edited the manuscript.

## Conflicts of interest

There are no conflicts to declare.

## Supplementary Material

SC-015-D3SC05353A-s001
