# Supplementary material for: Machine learning from quantum chemistry to predict experimental solvent effects on reaction rates
Source: Chem Sci. 2024 Jan 10;15(7):2410–24. doi: 10.1039/d3sc05353a (PMC10866337; doi:10.1039/d3sc05353a)
Supplement: SC-015-D3SC05353A-s001 [file SC-015-D3SC05353A-s001.pdf]

Electronic Supplementary Material (ESI) for Chemical Science.
This journal is © The Royal Society of Chemistry 2024

Supporting Information for:

Machine learning from quantum chemistry to predict experimental solvent effects on reaction rates

Yunsie Chung[†] and William H. Green[*,†]

[†]*Department of Chemical Engineering, Massachusetts Institute of Technology, Cambridge, MA, 02139, U.S.A*

E-mail: whgreen@mit.edu

S1 Details on the pre-training data set

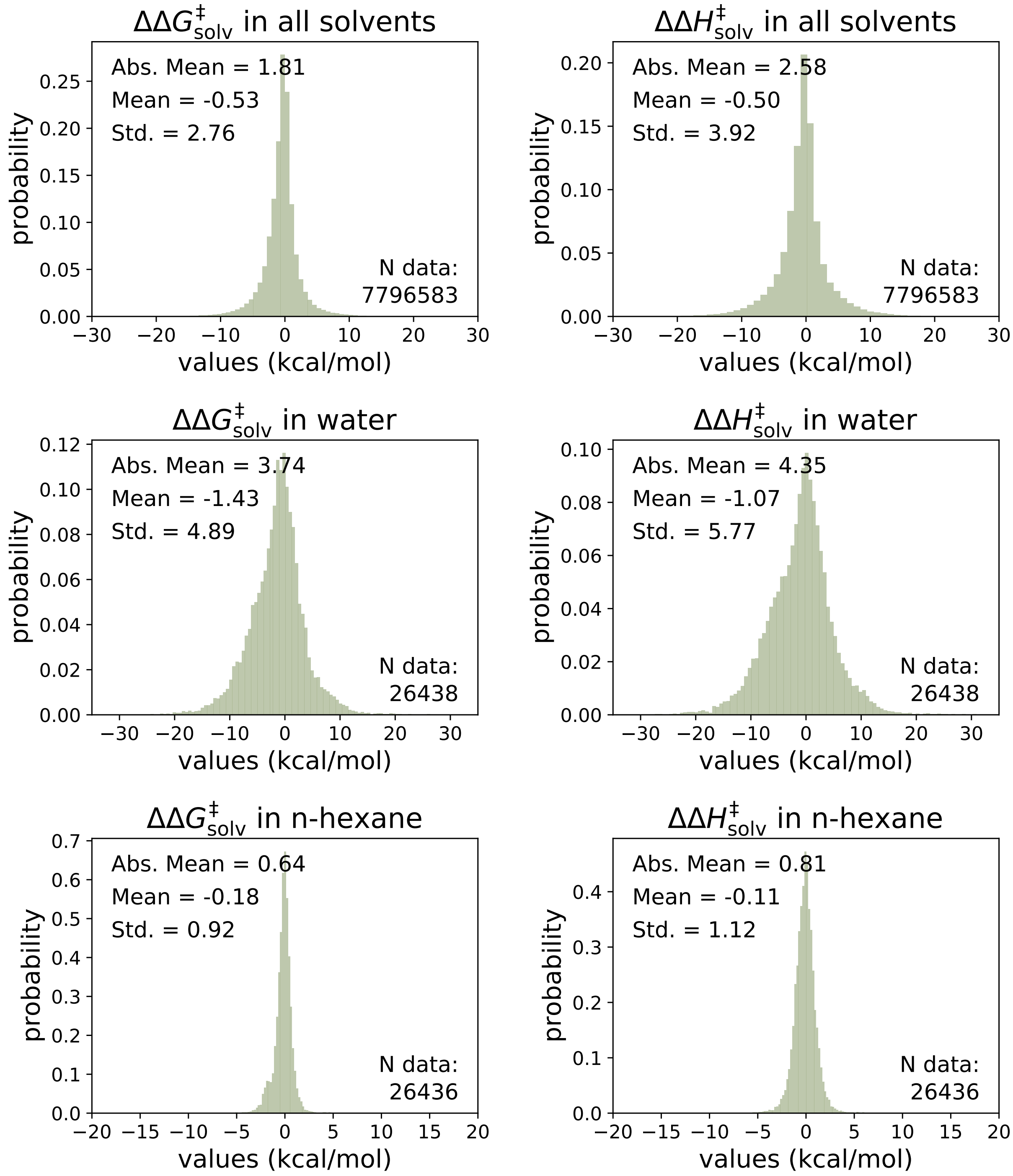

Figure S1: Distributions of the pre-training data set for the reactions in all solvents (top), in water (middle), and in n-hexane (bottom). The absolute mean (abs. mean), mean and standard deviation (std.) are in kcal/mol. Water and n-hexane are chosen as sample solvents for data visualization.

S2 Details on the fine-tuning data set

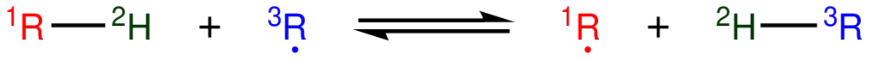

(a) Bimolecular hydrogen abstraction

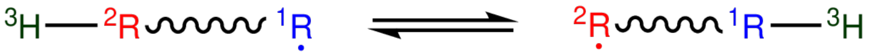

(b) Unimolecular hydrogen migration

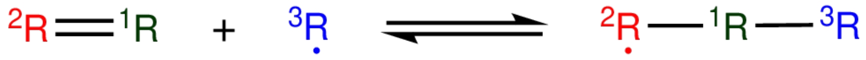

(c) Radical addition to a multiple bond

Figure S2: Templates of the reaction types found in the fine-tuning set.

A subset of the fine-tuning data set was selected for the model training. Around 25 solvents were sampled per each reaction with following procedure:

- Water, acetic acid, and acetonitrile are always included.
- Randomly sample 17 solvents from the following list of 44 more common solvents: carbon tetrachloride, benzene, tert-Butanol, hexane, anisole, T-amyl alcohol, ethyl acetate, propionitrile, chlorobenzene, methylformate, dioxane, tetrahydrofuran, tetrachloroethylene, bromobenzene, benzonitrile, cyclohexane, diethyl ether, methanol, 2-propanol, dichloromethane, chloroform, sulfolane, DMF, ethanol, 2,2,2-trifluoroethanol, acetone, toluene, isopropyl ether, veratrole, nitrobenzene, nitromethane, triethylene glycol, N-benzylacetamide, formamide, ethylene glycol, 2-methoxyethanol, benzyl alcohol, 2-phenoxyethanol, N-methylformamide, dimethyl sulfoxide, glycerol, isooctane, 3-methyl-3-pentanol, and 1-butanol.
- Sample 5 solvents from the remaining solvents.

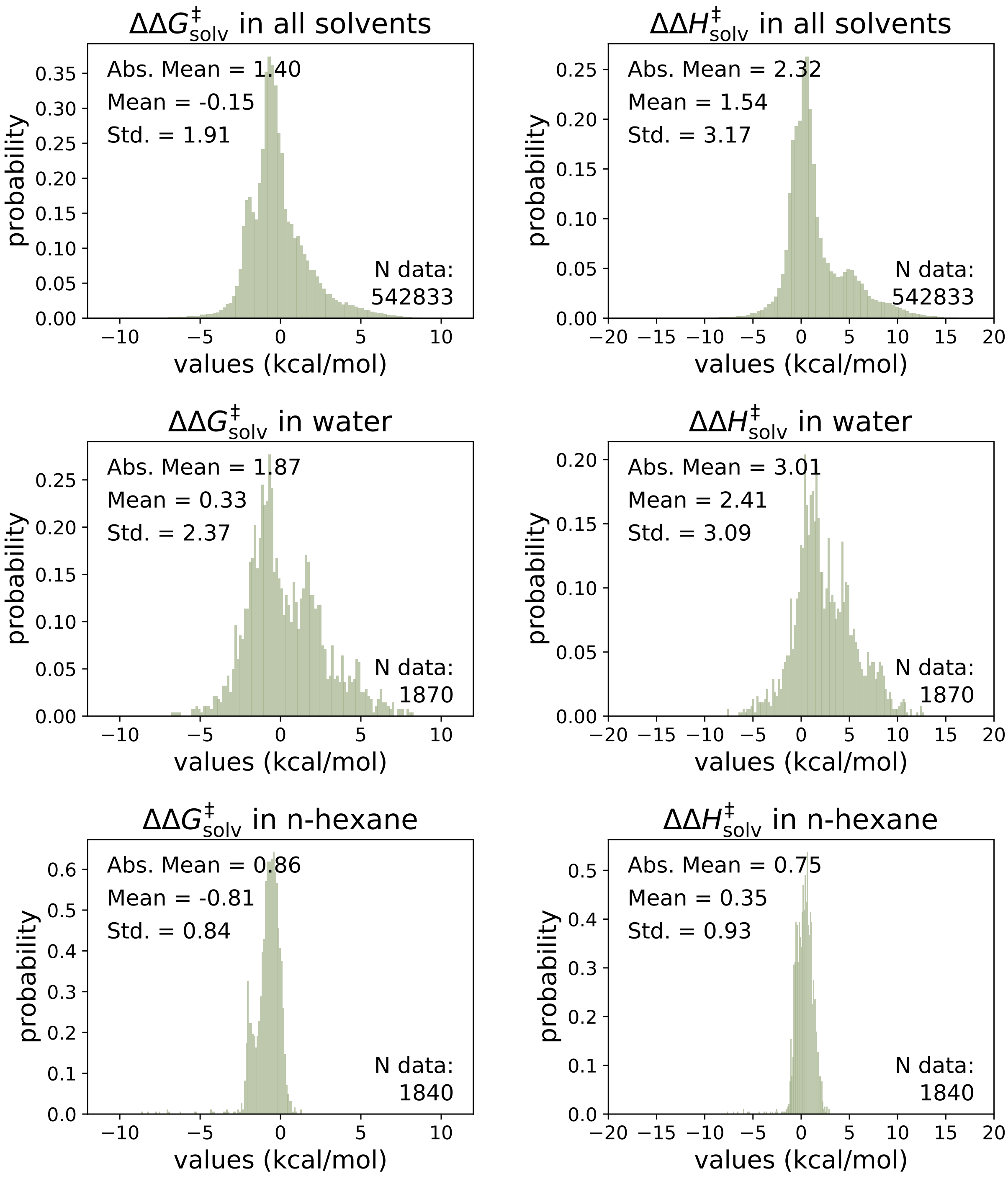

Figure S3: Distributions of the fine-tuning data set for the reactions in all solvents (top), in water (middle), and in n-hexane (bottom). The absolute mean (abs. mean), mean and standard deviation (std.) are in kcal/mol. Water and n-hexane are chosen as sample solvents for data visualization.

S3 Reactions in the experimental set

Table S1: List of reactions found in the experimental set.

Index	Reaction
1	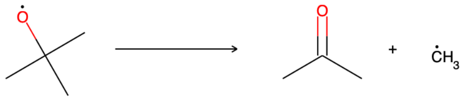<br>β-scission
2	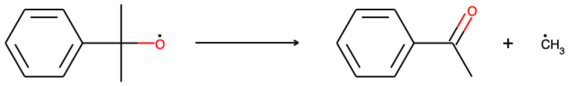<br>β-scission
3	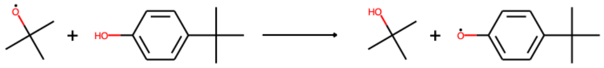<br>H-abstraction
4	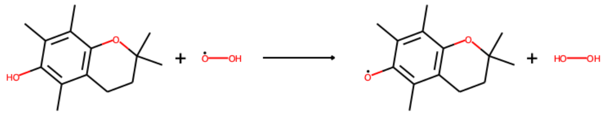<br>H-abstraction
5	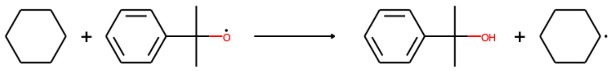<br>H-abstraction
6	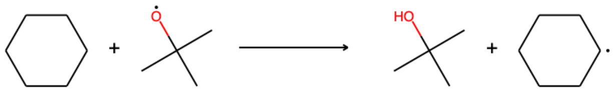<br>H-abstraction [a]
7	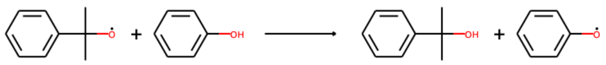<br>H-abstraction

8	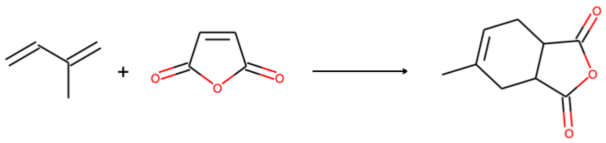<br>Diels–Alder
9	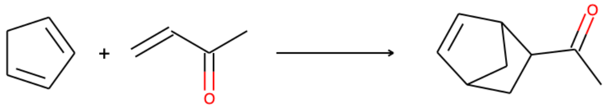<br>Diels–Alder
10	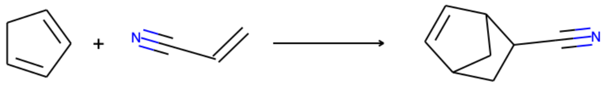<br>Diels–Alder
11	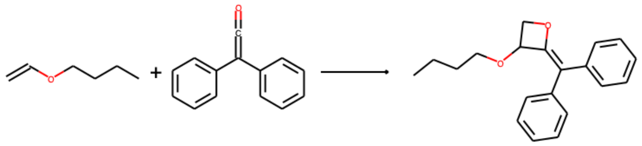<br>[2+2] Cycloaddition
12	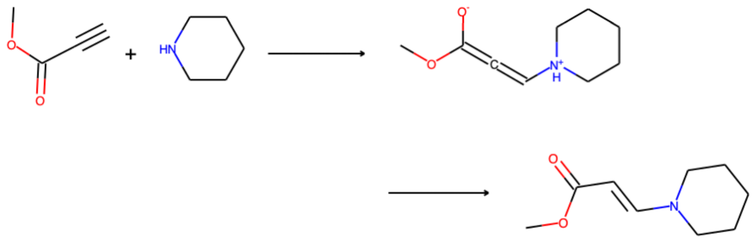<br>Nucleophilic addition to carbon-carbon triple bond [b]
13	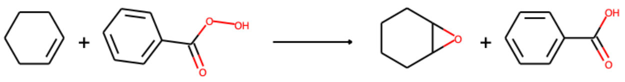<br>Epoxidation of cyclohexene with peroxycarboxylic acid
14	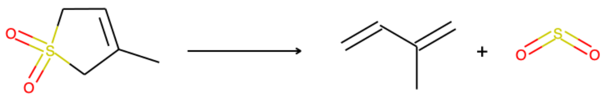<br>Thermal decomposition of 3-sulfolene

15	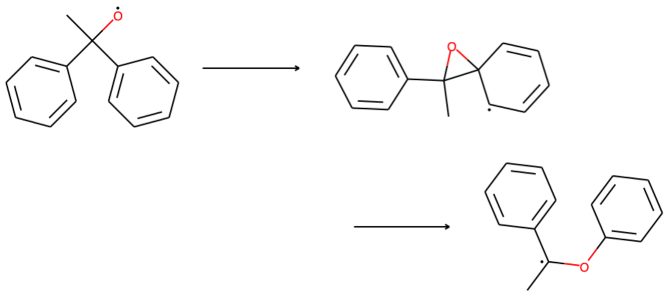 *O*-Neophyl rearrangement of 1,1-diarylalkoxyl radical [b]

[a] The rates constants of Reaction 6 are reported relative to the rate constants of Reaction 1. [b] The first step was used for the ML predictions for these reactions as the first step was previously identified as a rate limiting step.

S4 Details on the ML model

Table S2: Default atom and bond features used for the model.

**Atom features**	**Type**	**Bond features**	**Type**
Atomic number	One-hot	Bond type	One-hot
Number of bonds	One-hot	Conjugation	One-hot
Formal charge	One-hot	In ring	One-hot
Ring size	One-hot	Stereo	One-hot
Connected hydrogen atoms	One-hot		
Hybridization	One-hot		
Aromaticity	One-hot		
Molar mass	Numeric		

Table S3: Optimized hyperparameters.

Type	Optimized value
D-MPNN hidden size	Reaction: 900, Solvent: 200
D-MPNN depth	Reaction: 4, Solvent: 2
D-MPNN bias	No
Aggregation	Normalized
FNN hidden size	400
FNN depth	6
FNN bias	Yes
Activation function	ReLu
Dropout	0.0
Batch size	30
Warm-up epochs	4
Max epochs for pre-training	80
Max epochs for fine-tuning	10
Learning rates (initial, max, final)	1.8e-4, 2.2e-4, 5.2e-5

S5 Results on the pre-training set

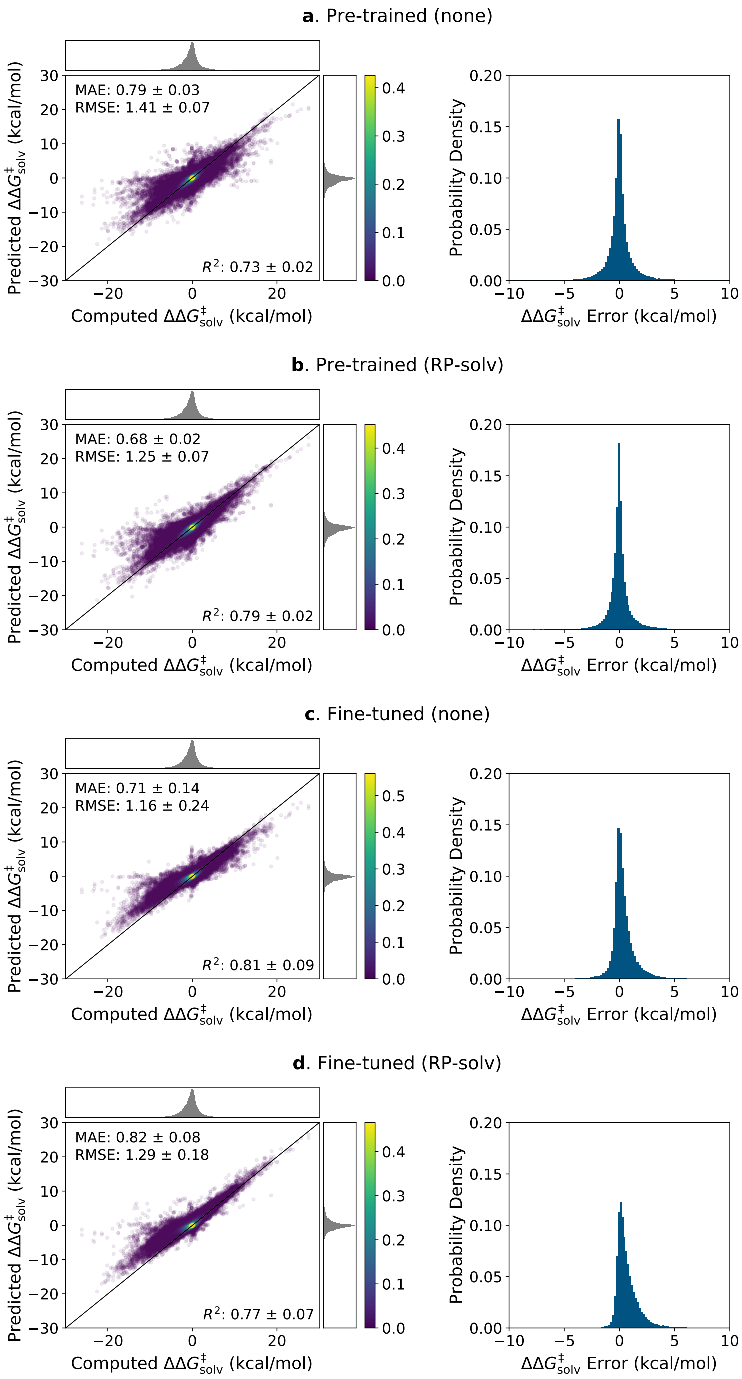

Figure S4: Parity plots and histograms of the $\Delta\Delta G^{\ddagger}_{\text{solv}}$ errors on the 5-fold pre-training set reaction split. (a) The pre-trained model with no additional feature. (b) The pre-trained model with the RP-solv feature. (c) The fine-tuned model with no additional feature. (d) The fine-tuned model with the RP-solv feature. The MAE and RMSE are in kcal/mol. The numbers of reactions, solvents, total data points found in the test set are provided.

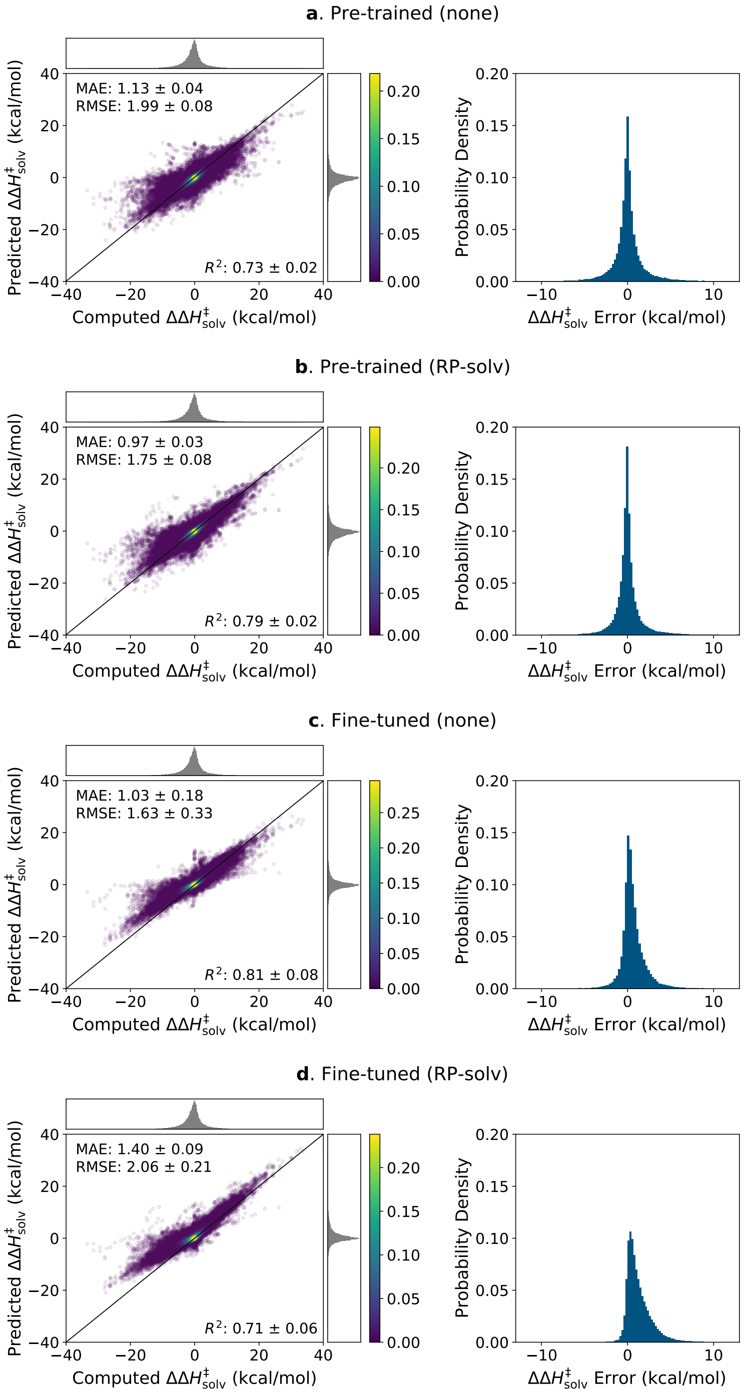

Figure S5: Parity plots and histograms of the $\Delta\Delta H^{\ddagger}_{\mathrm{solv}}$ errors on the 5-fold pre-training set reaction split. (a) The pre-trained model with no additional feature. (b) The pre-trained model with the RP-solv feature. (c) The fine-tuned model with no additional feature. (d) The fine-tuned model with the RP-solv feature. The MAE and RMSE are in kcal/mol. The numbers of reactions, solvents, total data points found in the test set are provided.

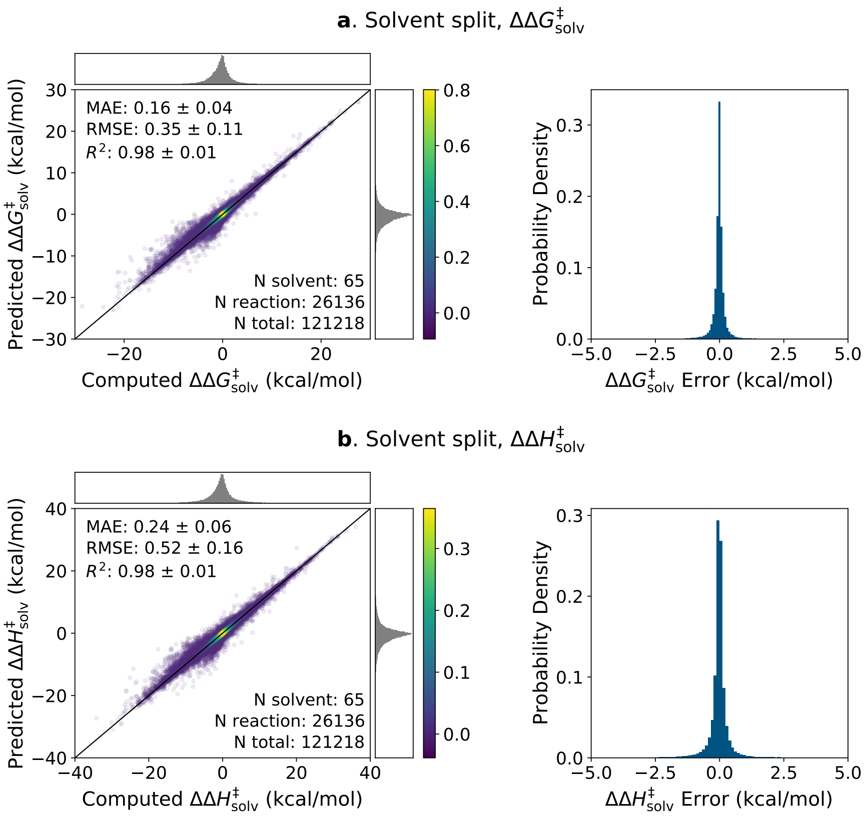

Figure S6: Parity plots and histograms of the test errors on the 5-fold pre-training set solvent split. The results correspond to the pre-trained model with the RP-solv feature. (a) $\Delta\Delta G^{\ddagger}_{\text{solv}}$ results. (b) $\Delta\Delta H^{\ddagger}_{\text{solv}}$ results. The MAE and RMSE are in kcal/mol. The numbers of reactions, solvents, total data points found in the test set are provided.

Table S4: Top 20 reactions with the highest RMSE on the pre-training test set with the reaction split. The test errors are computed with the fine-tuned model with no additional feature. The RMSE, mean $\Delta\Delta G^{\ddagger}_{\text{solv}}$ of the reaction across different solvents, and gas phase barrier height ($E_{a,\text{ gas}}$) are shown in kcal/mol for both forward and reverse directions. The gas phase barrier heights are obtained from the published works by Grambow et el.[1;2;3]

Rank	Reaction
1	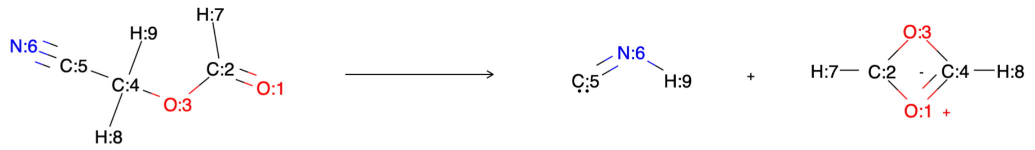 **Forward**: RMSE = 10.39, Mean $\Delta\Delta G^{\ddagger}_{\text{solv}}$ = -15.13 $E_{a,\text{ gas}}$ = 106.82 **Reverse**: RMSE = 8.46, Mean $\Delta\Delta G^{\ddagger}_{\text{solv}}$ = -11.48 $E_{a,\text{ gas}}$ = 11.27
2	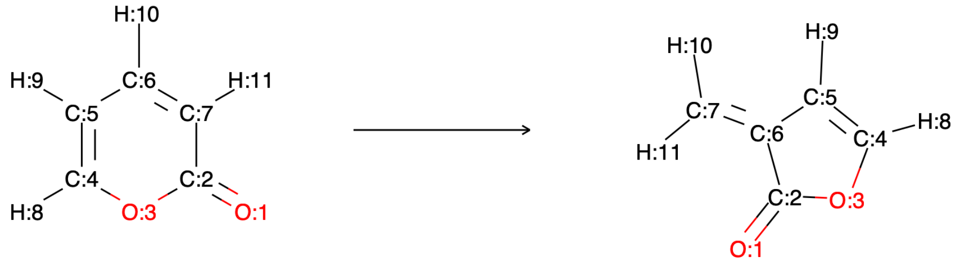 **Forward**: RMSE = 8.07, Mean $\Delta\Delta G^{\ddagger}_{\text{solv}}$ = -7.29 $E_{a,\text{ gas}}$ = 118.41 **Reverse**: RMSE = 8.28, Mean $\Delta\Delta G^{\ddagger}_{\text{solv}}$ = -7.61 $E_{a,\text{ gas}}$ = 109.81
3	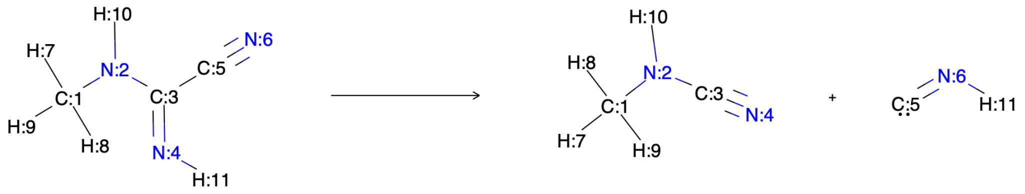 **Forward**: RMSE = 8.04, Mean $\Delta\Delta G^{\ddagger}_{\text{solv}}$ = -9.73 $E_{a,\text{ gas}}$ = 61.96 **Reverse**: RMSE = 5.26, Mean $\Delta\Delta G^{\ddagger}_{\text{solv}}$ = -3.68 $E_{a,\text{ gas}}$ = 37.76
4	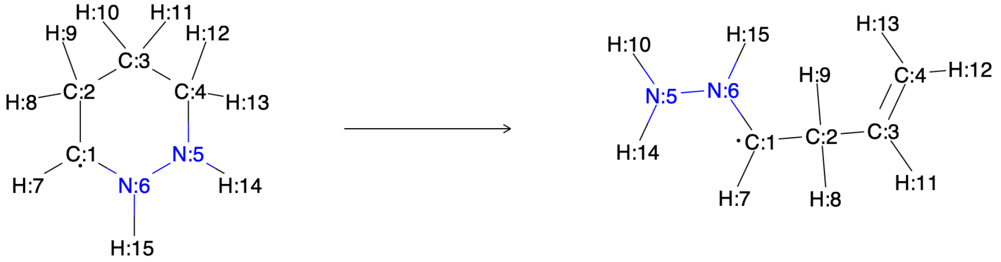 **Forward**: RMSE = 5.39, Mean $\Delta\Delta G^{\ddagger}_{\text{solv}}$ = -4.70 $E_{a,\text{ gas}}$ = 73.75 **Reverse**: RMSE = 7.78, Mean $\Delta\Delta G^{\ddagger}_{\text{solv}}$ = -7.34 $E_{a,\text{ gas}}$ = 64.63

5

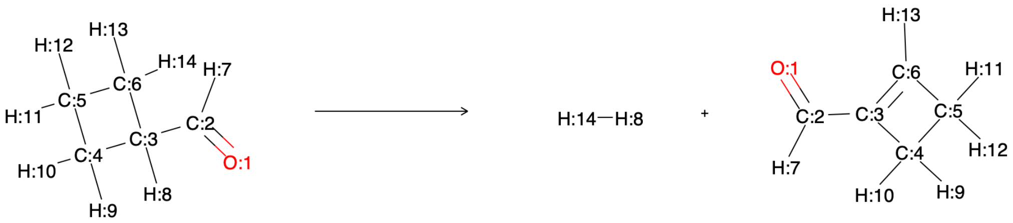

Forward: RMSE = 6.96, Mean $\Delta\Delta G^{\ddagger}_{\text{solv}}$ = -7.34 $E_{a,\text{ gas}}$ = 100.61
Reverse: RMSE = 7.62, Mean $\Delta\Delta G^{\ddagger}_{\text{solv}}$ = -9.66 $E_{a,\text{ gas}}$ = 69.47

6

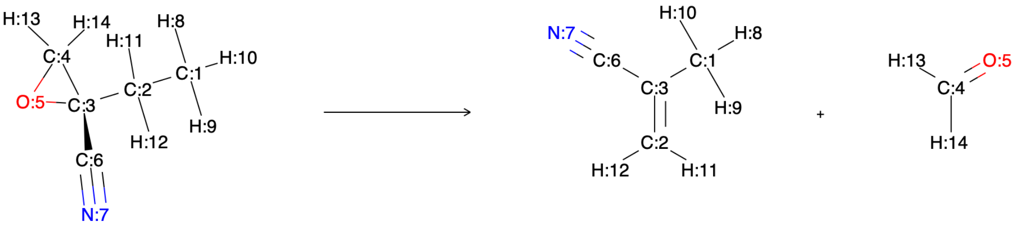

Forward: RMSE = 6.18, Mean $\Delta\Delta G^{\ddagger}_{\text{solv}}$ = -7.94 $E_{a,\text{ gas}}$ = 91.40
Reverse: RMSE = 6.67, Mean $\Delta\Delta G^{\ddagger}_{\text{solv}}$ = -9.90 $E_{a,\text{ gas}}$ = 90.10

7

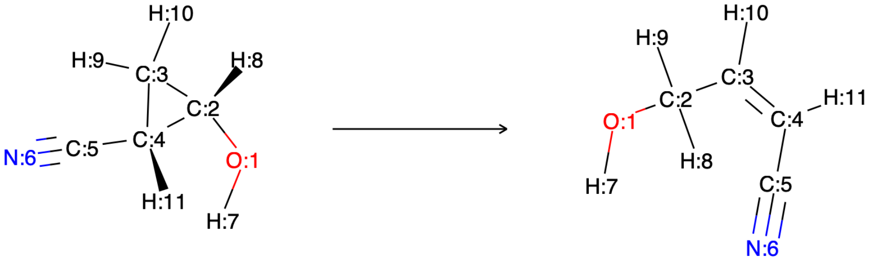

Forward: RMSE = 4.54, Mean $\Delta\Delta G^{\ddagger}_{\text{solv}}$ = -5.39 $E_{a,\text{ gas}}$ = 64.93
Reverse: RMSE = 6.51, Mean $\Delta\Delta G^{\ddagger}_{\text{solv}}$ = -8.10 $E_{a,\text{ gas}}$ = 68.22

8

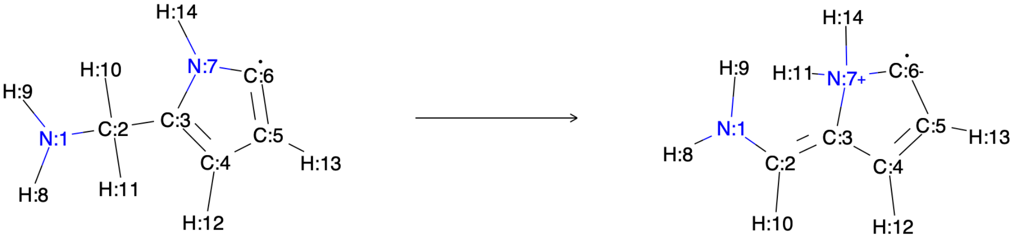

Forward: RMSE = 6.51, Mean $\Delta\Delta G^{\ddagger}_{\text{solv}}$ = -7.66 $E_{a,\text{ gas}}$ = 82.50
Reverse: RMSE = 0.73, Mean $\Delta\Delta G^{\ddagger}_{\text{solv}}$ = 3.63 $E_{a,\text{ gas}}$ = 48.45

<table>
<tr><td>9</td><td>
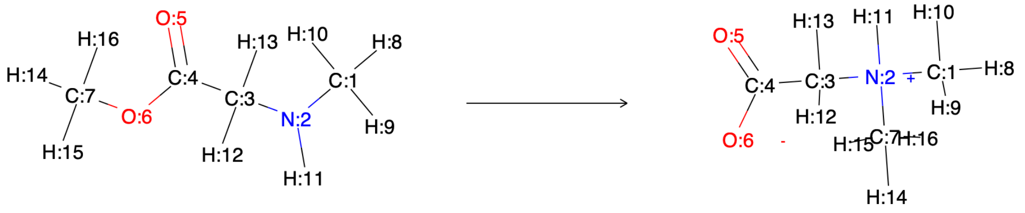

Forward: RMSE = 4.92, Mean $\Delta\Delta G^{\ddagger}_{\text{solv}}$ = -7.85 $E_{a,\text{ gas}} = 67.94$
Reverse: RMSE = 6.49, Mean $\Delta\Delta G^{\ddagger}_{\text{solv}}$ = 8.41 $E_{a,\text{ gas}} = 47.89$
</td></tr>
<tr><td>10</td><td>
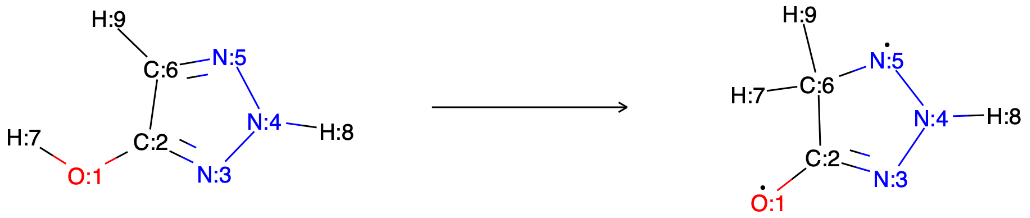

Forward: RMSE = 5.88, Mean $\Delta\Delta G^{\ddagger}_{\text{solv}}$ = -3.05 $E_{a,\text{ gas}} = 82.90$
Reverse: RMSE = 4.58, Mean $\Delta\Delta G^{\ddagger}_{\text{solv}}$ = -2.10 $E_{a,\text{ gas}} = 58.42$
</td></tr>
<tr><td>11</td><td>
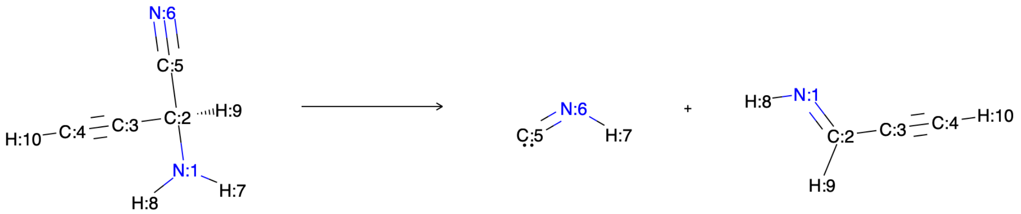

Forward: RMSE = 5.82, Mean $\Delta\Delta G^{\ddagger}_{\text{solv}}$ = -11.18 $E_{a,\text{ gas}} = 56.95$
Reverse: RMSE = 4.03, Mean $\Delta\Delta G^{\ddagger}_{\text{solv}}$ = -7.00 $E_{a,\text{ gas}} = 35.19$
</td></tr>
<tr><td>12</td><td>
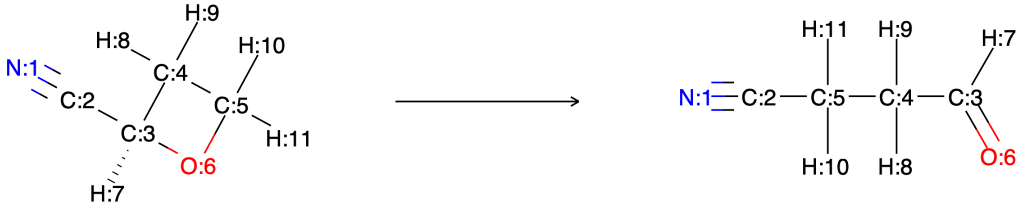

Forward: RMSE = 5.81, Mean $\Delta\Delta G^{\ddagger}_{\text{solv}}$ = -6.83 $E_{a,\text{ gas}} = 95.34$
Reverse: RMSE = 5.33, Mean $\Delta\Delta G^{\ddagger}_{\text{solv}}$ = -6.12 $E_{a,\text{ gas}} = 119.94$
</td></tr>
</table>

13	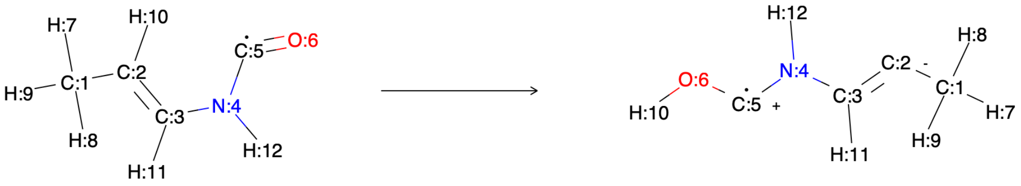<br>**Forward:** RMSE = 3.39, Mean $\Delta\Delta G^{\ddagger}_{\text{solv}}$ = -2.42 $E_{a,\text{ gas}}$ = 63.52<br>**Reverse:** RMSE = 5.78, Mean $\Delta\Delta G^{\ddagger}_{\text{solv}}$ = -0.22 $E_{a,\text{ gas}}$ = 2.80
14	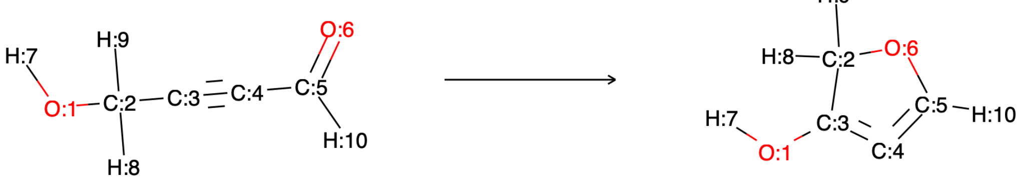<br>**Forward:** RMSE = 5.68, Mean $\Delta\Delta G^{\ddagger}_{\text{solv}}$ = -6.69 $E_{a,\text{ gas}}$ = 87.57<br>**Reverse:** RMSE = 2.40, Mean $\Delta\Delta G^{\ddagger}_{\text{solv}}$ = -3.05 $E_{a,\text{ gas}}$ = 73.83
15	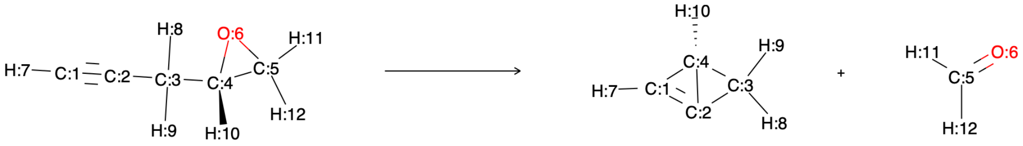<br>**Forward:** RMSE = 5.61, Mean $\Delta\Delta G^{\ddagger}_{\text{solv}}$ = -6.09 $E_{a,\text{ gas}}$ = 77.90<br>**Reverse:** RMSE = 4.87, Mean $\Delta\Delta G^{\ddagger}_{\text{solv}}$ = -5.28 $E_{a,\text{ gas}}$ = 30.34
16	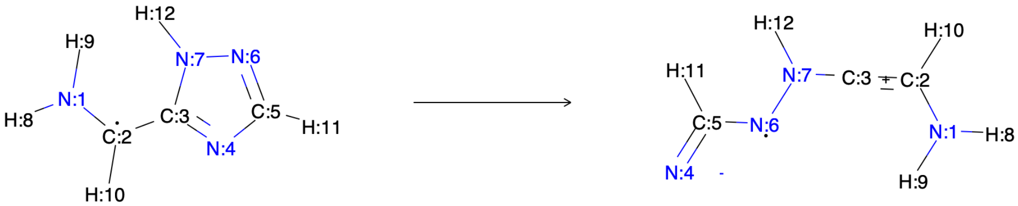<br>**Forward:** RMSE = 5.43, Mean $\Delta\Delta G^{\ddagger}_{\text{solv}}$ = -4.63 $E_{a,\text{ gas}}$ = 105.63<br>**Reverse:** RMSE = 4.57, Mean $\Delta\Delta G^{\ddagger}_{\text{solv}}$ = -3.52 $E_{a,\text{ gas}}$ = 8.89

17	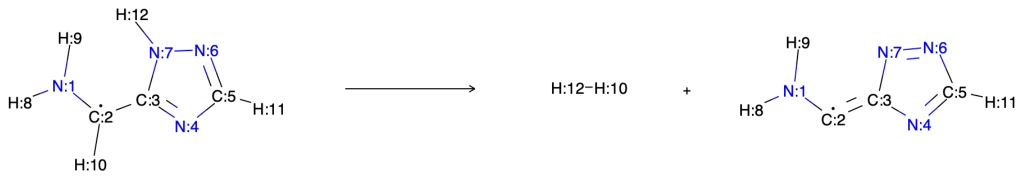<br>**Forward**: RMSE = 5.29, Mean $\Delta\Delta G^{\ddagger}_{\text{solv}}$ = -4.12 $E_{a,\text{ gas}}$ = 76.20<br>**Reverse**: RMSE = 0.35, Mean $\Delta\Delta G^{\ddagger}_{\text{solv}}$ = -0.82 $E_{a,\text{ gas}}$ = 5.24
18	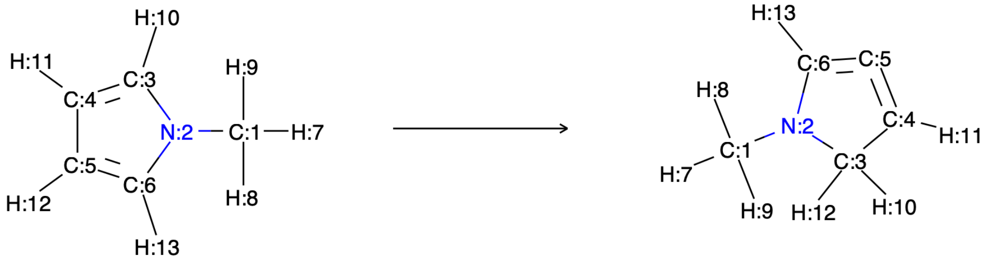<br>**Forward**: RMSE = 5.24, Mean $\Delta\Delta G^{\ddagger}_{\text{solv}}$ = -11.53 $E_{a,\text{ gas}}$ = 93.04<br>**Reverse**: RMSE = 0.31, Mean $\Delta\Delta G^{\ddagger}_{\text{solv}}$ = 0.28 $E_{a,\text{ gas}}$ = 22.60
19	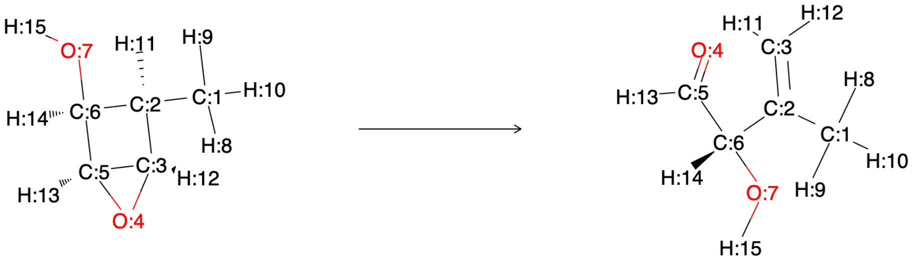<br>**Forward**: RMSE = 3.74, Mean $\Delta\Delta G^{\ddagger}_{\text{solv}}$ = -6.78 $E_{a,\text{ gas}}$ = 58.09<br>**Reverse**: RMSE = 5.23, Mean $\Delta\Delta G^{\ddagger}_{\text{solv}}$ = -7.67 $E_{a,\text{ gas}}$ = 83.93
20	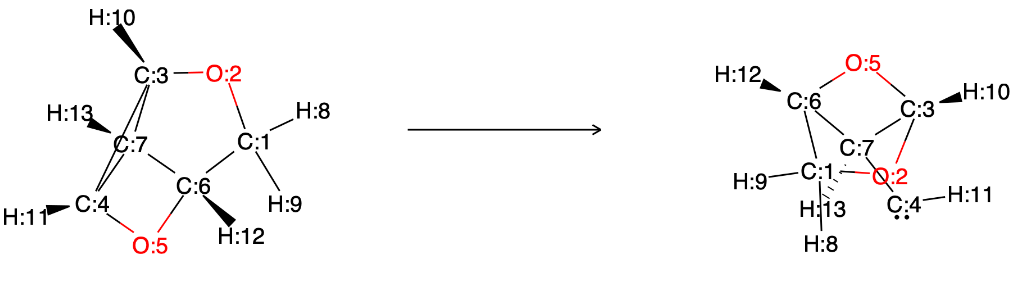<br>**Forward**: RMSE = 5.15, Mean $\Delta\Delta G^{\ddagger}_{\text{solv}}$ = -6.35 $E_{a,\text{ gas}}$ = 86.67<br>**Reverse**: RMSE = 4.36, Mean $\Delta\Delta G^{\ddagger}_{\text{solv}}$ = -3.85 $E_{a,\text{ gas}}$ = 18.28

Table S5: Examples of reactions found in the 10 most frequent reaction types

1. +C-H,-C-H,-C-C (647 reactions)

SMARTS: [C:1].[C:2][H:3]»[C:2][C:1][H:3]

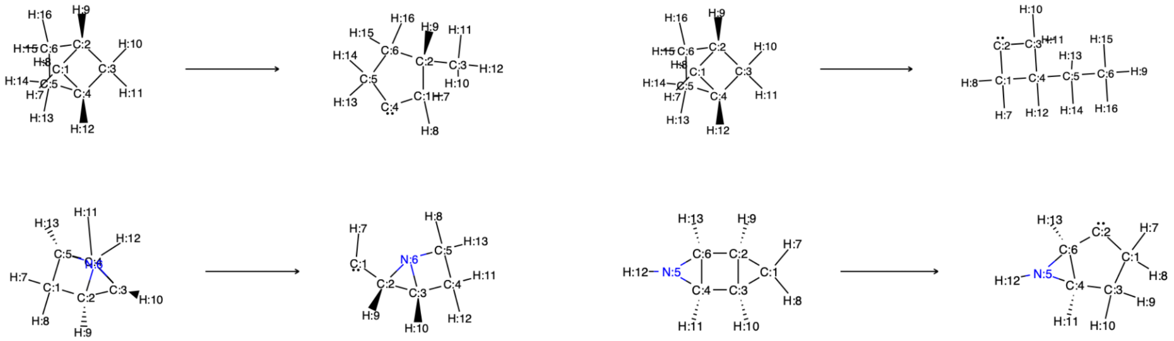

2. +O-H,+C=C,-C-O,-C-H (224 reactions)

SMARTS: [C:1]=[C:2].[O:3][H:4]»[C:1]([C:2][O:3])[H:4]

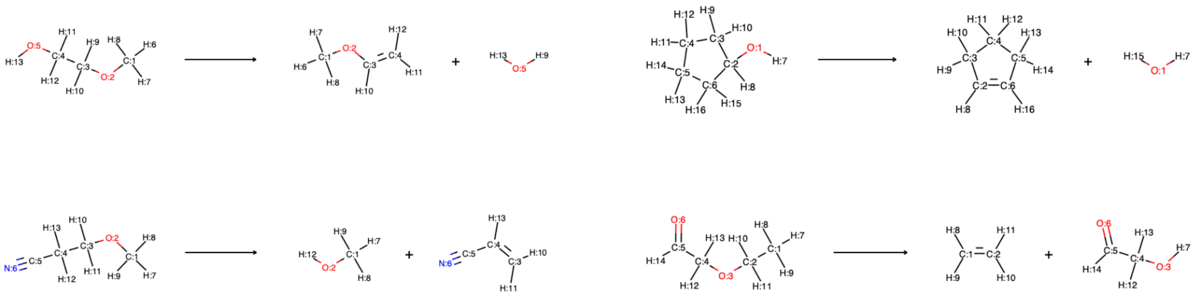

3. +C=C,+H-H,-C-H,-C-H (183 reactions)

SMARTS: [C:1]=[C:2].[H:3][H:4]»[C:1]([H:3])[C:2][H:4]

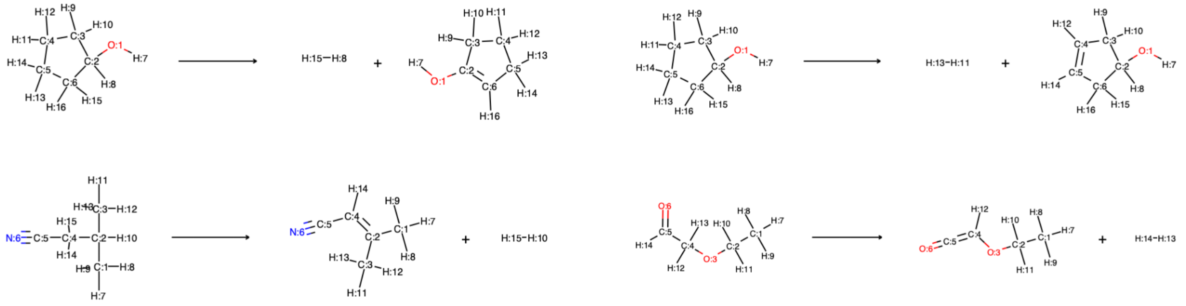

4. +C-O,-C-O,-C-C (133 reactions)

SMARTS: [C:1].[C:2][O:3]»[C:2][C:1][O:3]

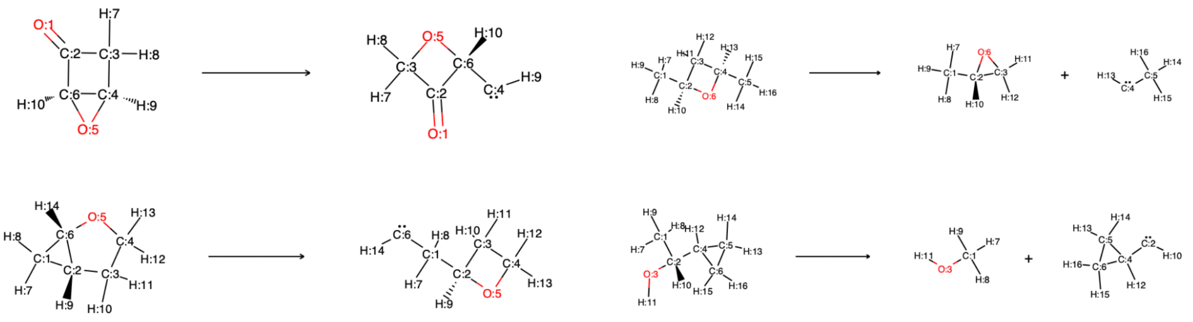

5. +C-C#N,-C-N#C (110 reactions)

SMARTS: [C:1][N+:2]#[C-:3]»[C:1][C:3]#[N:2]

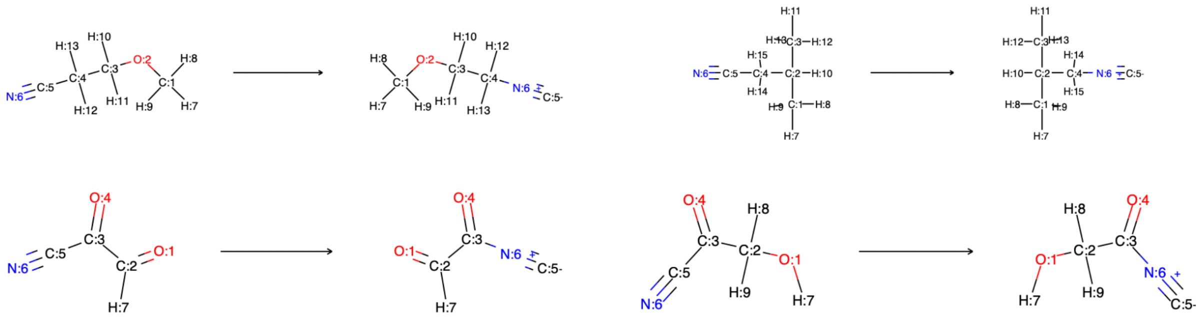

6. +C-C,-C-C,-C-C (107 reactions)

SMARTS: [C:1].[C:2][C:3]»[C:2][C:1][C:3]

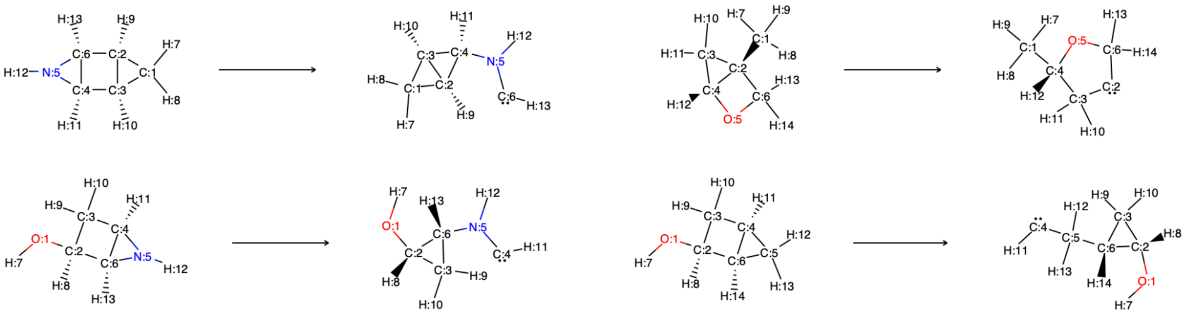

7. +O-H,-C-O,-C-H (107 reactions)

SMARTS: [C:1].[O:2][H:3]»[C:1]([H:3])[O:2]

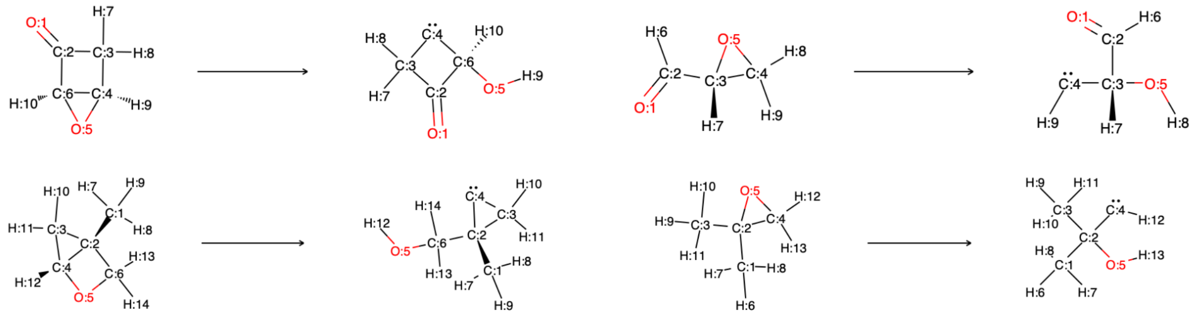

8. +C=C,+C-H,-C-C,-C-H (94 reactions)

SMARTS: [C:1]=[C:2].[C:3][H:4]»[C:3][C:1][C:2][H:4]

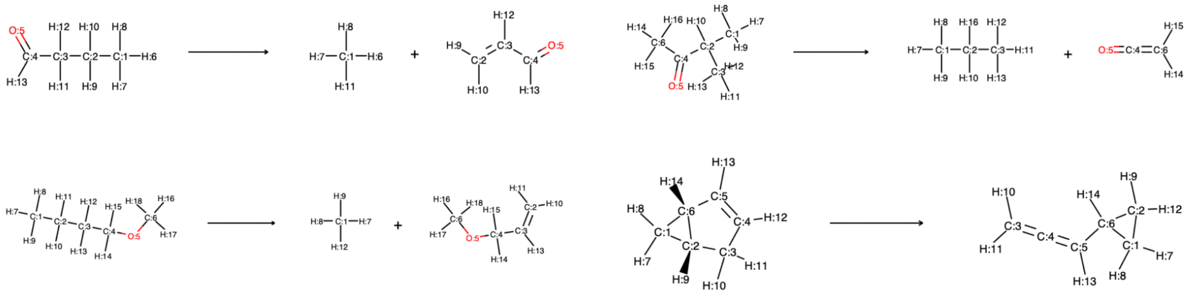

Top $\Delta\Delta G^{\ddagger}_{\text{solv}}$ error reactions:

MAE = 2.2 kcal/mol

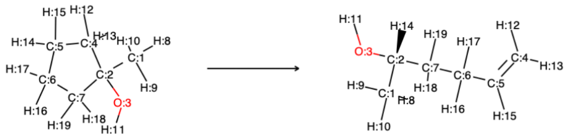

MAE = 1.8 kcal/mol

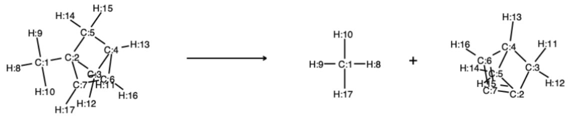

MAE = 1.7 kcal/mol

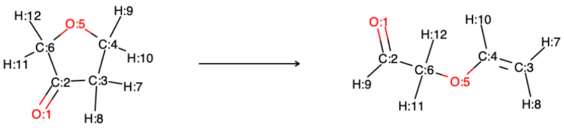

MAE = 1.6 kcal/mol

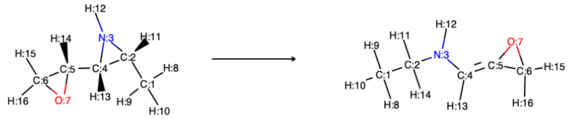

9. +C=O,+C-H,-C-O,-C-C,-C-H (87 reactions)

SMARTS: [C-:1]=[O+:2].[C:3][H:4]»[C:3][C:1]([H:4])[O:2]

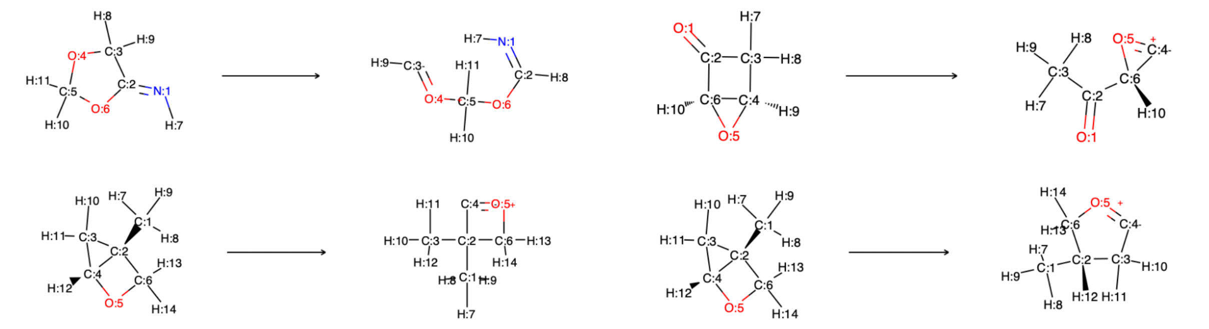

10. +C=C,+O-H,-C=O,C-H (79 reactions)

SMARTS: [C:1]=[C:2][O:3][H:4]»[C:1]([C:2]=[O:3])[H:4]

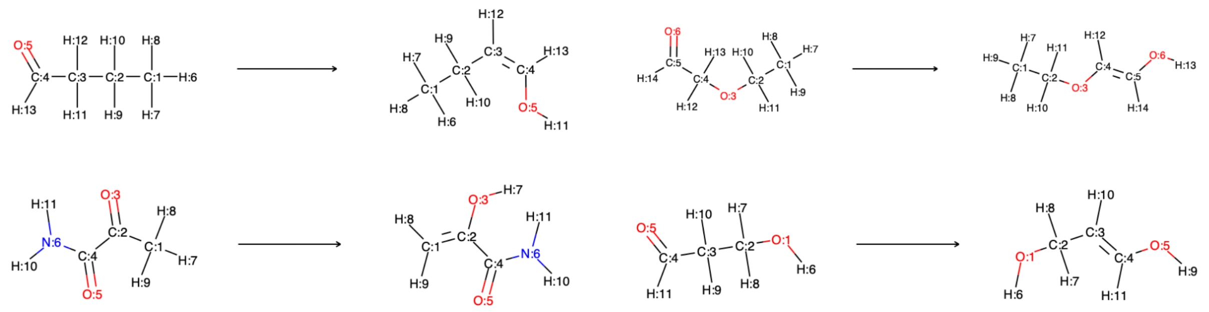

References

[1] Grambow, C. A.; Pattanaik, L.; Green, W. H. Reactants, products, and transition states of elementary chemical reactions based on quantum chemistry. *Sci. Data* **2020**, *7*.

[2] Grambow, C. A.; Pattanaik, L.; Green, W. H. Reactants, products, and transition states of elementary chemical reactions based on quantum chemistry (1.0.1) [Data set]. *Zenodo* **2020**, DOI: 10.5281/zenodo.3715478.

[3] Grambow, C. A. Reactants, products, and transition states of radical reactions (1.0.0) [Data set]. *Zenodo* **2020**, DOI: 10.5281/zenodo.3731554.
